# Multiorgan Dysfunction and Associated Prognosis in Transthyretin Cardiac Amyloidosis

**DOI:** 10.1161/JAHA.123.033094

**Published:** 2024-02-05

**Authors:** Adam Ioannou, Christian Nitsche, Aldostefano Porcari, Rishi K. Patel, Yousuf Razvi, Muhammad U. Rauf, Ana Martinez‐Naharro, Lucia Venneri, Antonella Accietto, Lucrezia Netti, Francesco Bandera, Ruta Virsinskaite, Tushar Kotecha, Dan Knight, Aviva Petrie, Carol Whelan, Ashutosh Wechalekar, Helen Lachmann, Philip N. Hawkins, Julian D. Gillmore, Marianna Fontana

**Affiliations:** ^1^ National Amyloidosis Centre University College London London United Kingdom; ^2^ Center for Diagnosis and Treatment of Cardiomyopathies, Cardiovascular Department Azienda Sanitaria Universitaria Giuliano‐Isontina, University of Trieste Trieste Italy; ^3^ University College London London United Kingdom; ^4^ Cardiology Unit, Department of Cardiac Thoracic and Vascular Istituto di Ricovero e Cura a Carattere Scientifico Azienda Ospedaliero–Universitaria di Bologna Bologna Italy; ^5^ Department of Clinical, Internal, Anesthesiological and Cardiovascular Sciences Sapienza University of Rome Rome Italy; ^6^ Heart Failure and Rehabilitation Unit, IRCCS MultiMedica, Sesto San Giovanni Milan Italy; ^7^ Department for Biomedical Sciences for Health University of Milano Milan Italy

**Keywords:** blood biomarkers, heart failure, mortality, transthyretin cardiac amyloidosis, Biomarkers, Cardiomyopathy, Heart Failure

## Abstract

**Background:**

Transthyretin cardiac amyloidosis (ATTR‐CA) is a progressive and ultimately fatal cardiomyopathy. Biomarkers reflecting multiorgan dysfunction are of increasing importance in patients with heart failure; however, their significance in ATTR‐CA remains largely unknown. The aims of this study were to characterize the multifaceted nature of ATTR‐CA using blood biomarkers and assess the association between blood biomarkers and prognosis.

**Methods and Results:**

This is a retrospective cohort study of 2566 consecutive patients diagnosed with ATTR‐CA between 2007 and 2023. Anemia (39%), high urea (52%), hyperbilirubinemia (18%), increased alkaline phosphatase (16%), increased CRP (C‐reactive protein; 27%), and increased troponin (98.2%) were common findings in the overall population, whereas hyponatremia (6%) and hypoalbuminemia (2%) were less common. These abnormalities were most common in patients with p.(V142I) hereditary ATTR‐CA, and became more prevalent as the severity of cardiac disease increased. Multivariable Cox regression analysis demonstrated that anemia (hazard ratio [HR], 1.19 [95% CI, 1.04–1.37]; *P*=0.01), high urea (HR, 1.23 [95% CI, 1.04–1.45]; *P*=0.01), hyperbilirubinemia (HR, 1.32 [95% CI, 1.13–1.57; *P*=0.001), increased alkaline phosphatase (HR, 1.20 [95% CI, 1.01–1.42; *P*=0.04), hyponatremia (HR, 1.65 [95% CI, 1.28–2.11]; *P*<0.001), and troponin‐T >56 ng/L (HR, 1.72 [95% CI, 1.46–2.03]; *P*<0.001) were all independently associated with mortality in the overall population. The association between biomarkers and mortality varied across the spectrum of genotypes and left ventricular ejection fraction, with anemia remining independently associated with mortality in p.(V142I) hereditary ATTR‐CA (HR, 1.58 [95% CI, 1.17–2.12]; *P*=0.003) and in a subgroup of the overall population with a left ventricular ejection fraction ≤40% (HR, 1.39 [95% CI, 1.08–1.81]; *P*=0.01).

**Conclusions:**

Cardiac and noncardiac biomarker abnormalities were common and reflect the complex and multifaceted nature of ATTR‐CA, with a wide range of biomarkers remaining independently associated with mortality. Clinical trials are needed to investigate whether biomarker abnormalities represent modifiable risk factors that if specifically targeted could improve outcomes.

**Nonstandard Abbreviations and Acronyms**
ALP: alkaline phosphatase
ATTR‐CA: transthyretin cardiac amyloidosis
hATTR‐CA: hereditary transthyretin cardiac amyloidosis
NAC: National Amyloidosis Centre
NYHA: New York Heart Association
wtATTR‐CA: wild‐type transthyretin cardiac amyloidosis

**Clinical Perspective**

**What Is New?**

- In this large cohort of patients with transthyretin cardiac amyloidosis, both cardiac and noncardiac blood biomarker abnormalities were common and were more prevalent in patients with p.(V142I) hereditary transthyretin cardiac amyloidosis and advanced cardiac disease.
- Transthyretin cardiac amyloidosis is a complex and multifaceted disease process, whereby multiple pathways involving different organs and systems, beyond cardiac infiltration, contribute to disease progression and prognosis.

**What Are the Clinical Implications?**

- Whether treatment aimed at reversing blood biomarker abnormalities in this population would potentially reduce the risk of mortality remains unknown; therefore, clinical trials are needed to investigate whether biomarker abnormalities represent modifiable risk factors that if specifically targeted could improve outcomes.

Transthyretin cardiac amyloidosis (ATTR‐CA) is an increasingly recognized cause of heart failure (HF). Transthyretin is a tetramer that is naturally synthesized by the liver, and it is the physiological transport protein for thyroxine and retinol. Disease occurs when the transthyretin protein misfolds into amyloid fibrils that deposit within the myocardial extracellular space. The accumulation of amyloid fibrils disrupts the cardiac structure and function, and causes a progressive and ultimately fatal cardiomyopathy.^1^ The sporadic, noninherited, wildtype ATTR‐CA (wtATTR‐CA) has become the most prevalent form, and presents later in life; whereas the hereditary form (hATTR‐CA) can present earlier in life with a varying clinical phenotype, often composed of both restrictive cardiomyopathy and polyneuropathy.^2^, ^3^

Diagnosis is made at varying disease stages, and although increased awareness among clinicians and improvements in diagnostics combined with the recognition of characteristic imaging features have facilitated earlier diagnosis, a significant proportion of patients are still diagnosed with advanced cardiac disease.^1^, ^4^ A well‐established staging system composed of NT‐proBNP (N‐terminal pro‐B‐type natriuretic peptide) and estimated glomerular filtration rate accurately stratifies patients into prognostic categories.^5^

Chronic HF is associated with an enduring inflammatory state, consisting of the upregulation of inflammatory cytokines and unending myocyte damage; the consequences of venous congestion include congestive cirrhosis,^6^ gut malabsorption, and subsequent iron deficiency and anemia.^7^ More important, biomarkers reflecting multiorgan dysfunction have been independently associated with a worse prognosis in the population with HF.^6^, ^7^, ^8^ Despite many studies characterizing the significance of noncardiac biomarkers in HF in general, the importance of these biomarkers in patients with HF in the setting of ATTR‐CA remains largely unknown.

The aims of this study were to: (1) characterize the multifaceted nature of ATTR‐CA using blood biomarkers that reflect both cardiac and noncardiac disease activity, across a spectrum of wtATTR‐CA and hATTR‐CA; (2) describe the different patterns of abnormalities in cardiac and noncardiac blood biomarkers across the spectrum of ATTR‐CA disease stages; and (3) assess the association between the different biomarkers and prognosis.

## Methods

The data that support the findings of this study are restricted by the institutional ethics committee to protect patient privacy, and therefore cannot be shared.

Consecutive patients diagnosed with ATTR‐CA between 2007 and 2023 at the National Amyloidosis Centre (NAC), and in whom a full blood cell count and serum biochemistry assessing renal function, liver function, and NT‐proBNP were measured at diagnosis were included. Blood biomarker normal ranges are displayed in Table S1. Data on ethnicity were self‐reported.

Between 2007 and 2010, the diagnosis of ATTR‐CA was established on the basis of HF symptoms together with a characteristic cardiac amyloidosis echocardiogram or cardiac magnetic resonance and either direct endomyocardial biopsy proof of ATTR‐amyloid or ATTR‐amyloid in an extracardiac biopsy. From 2010 onwards, ^99m^technetium‐labeled 3,3‐diphosphono‐1,2‐propanodicarboxylic acid scintigraphy was used, and a diagnosis established on the basis of ATTR‐amyloid in an extracardiac biopsy with cardiac uptake on ^99m^technetium‐labeled 3,3‐diphosphono‐1,2‐propanodicarboxylic acid scintigraphy; or grade 2 to 3 cardiac uptake on ^99m^technetium‐labeled 3,3‐diphosphono‐1,2‐propanodicarboxylic acid scintigraphy in the absence of biochemical evidence of a plasma cell dyscrasia. All patients underwent genetic sequencing of the *TTR* gene and provided written consent for their data to be retrospectively analyzed and published, in line with the Declaration of Helsinki and approval from the Royal Free Hospital ethics committee (REC 21/PR/0620).

### Statistical Analysis

Statistical analysis was performed using Stata (StataCorp, 2021, Stata Statistical Software: Release 17). All continuous variables were tested for normality (Shapiro‐Wilk test) and presented as mean±SD if the distribution was normal or median (interquartile range) otherwise. The independent samples *t‐*test was used to compare means if the data were normally distributed, or its nonparametric equivalent to compare the distributions of the 2 groups. One‐way ANOVA if the data were normally distributed in each group was used to compare means; or its nonparametric equivalent to compare the distributions of multiple groups. A significant result was followed by post hoc Bonferroni corrected pairwise comparisons to establish where differences lay. Standardized differences were also calculated to demonstrate the effect size between groups. Categorical data are presented as absolute numbers (n) and frequencies (%) and compared using the χ^2^ test.

Correlations between stroke volume and blood biomarkers were assessed using Spearman ρ. A multivariable linear regression model was used to assess the relationship between stroke volume and blood biomarkers.

All mortality data were obtained via the UK Office of National Statistics. The mortality end point was defined as time to death from baseline for all deceased patients and time to censor date (March 17, 2023) from baseline among the remainder. Follow‐up was restricted to ≤60 months, after which patients were censored because most events occurred in the first 60 months, and a low number of patients at risk after 60 months.

Blood biomarkers were assessed as continuous variables, z‐score standardized variables, and dichotomous variables (normal versus abnormal), except for troponin, whereby the optimal cutoff was established using a time‐dependant receiver operating characteristic curve, followed by the Liu method.^9^ The optimal cut point was 58 ng/L (sensitivity, 64.9%; specificity, 66.3%). A cut point of 56 ng/L (4×upper limit of normal) gave a sensitivity of 63.7% and a specificity of 67.2%, and was selected for the survival analysis. Univariable Cox proportional hazards regression was used to assess the association between blood biomarkers and mortality. The proportional hazards assumption was checked and confirmed. The likelihood ratio test was used to evaluate the contribution of adding explanatory variables to the NAC disease stage. Multivariable Cox regression models were adjusted for known predictors of mortality and blood biomarkers selected a priori for clinical relevance. Possible collinearity among candidate predictors was assessed using variance inflation factors with threshold equal to 3. To account for the effect of amyloid‐specific disease‐modifying therapy or clinical trials, a second multivariable model was created as a sensitivity analysis, whereby patients were censored at their start date. Harrell's C statistic was calculated for the different models. Kaplan‐Meier curves were constructed with statistical significance being assessed with a log‐rank test. Statistical significance was defined as *P*<0.05.

## Results

We identified 2566 patients diagnosed with ATTR‐CA. The population comprised 1834 (71.5%) with wtATTR‐CA, 425 (16.6%) with p.(V142I) hATTR‐CA, and 307 (12.0%) with non‐p.(V142I) hATTR‐CA. The mean age was 76.9±8.2 years, and 86.4% were men. When compared with wtATTR, patients with hATTR‐CA were younger, and a higher proportion were women. Most patients with wtATTR‐CA and non‐p.(V142I) hATTR‐CA were White, whereas most patients with p.(V142I) hATTR‐CA were Black (Table 1).

**Table 1:** Baseline Characteristics and Blood Biomarkers for the Whole Population With ATTR‐CA, and Split by Genotype Into Patients With wtATTR‐CA, p.(V142I) hATTR‐CA, and Non‐p.(V142I) hATTR‐CA

Characteristic	Overall population (n=2566)	wtATTR‐CA (n=1834, 71.5%)	p.(V142I) hATTR‐CA (n=425, 16.6%)	Non‐p.(V142I) hATTR‐CA (n=307, 12.0%)	*P* value
Demographics					
Age, y	76.9±8.2	78.8±6.6^*†^	76.4±7.3^‡^	66.4±9.8	<0.001
Male sex	2218 (86.4)	1715 (93.5)^*†^	288 (67.8)	215 (70.0)	<0.001
Race					<0.001
White	2034 (79.3)	1727 (94.2)^*†^	40 (9.4)^‡^	267 (87.0)	
Black	494 (19.3)	91 (5.0)*	378 (88.9)^‡^	25 (8.1)	
Asian	38 (1.5)	16 (0.9)^†^	7 (1.6)	15 (4.9)	
Comorbidities					
Ischemic heart disease	483 (18.8)	409 (22.3)^*†^	57 (13.4)^‡^	17 (5.5)	<0.001
Hypertension	847 (33.0)	591 (32.2)^*†^	213 (50.1)^‡^	43 (14.0)	<0.001
Diabetes	390 (15.2)	266 (14.5)^*†^	103 (26.4)^‡^	21 (5.4)	<0.001
Stroke/TIA	265 (10.3)	201 (11.0)*	51 (12.0)^‡^	13 (4.2)	0.001
Atrial fibrillation	1254 (48.9)	1019 (55.6)^*†^	146 (34.4)	307 (29.0)	<0.001
ATTR‐CA diagnosis					
Cardiac biopsy	383 (14.9)	282 (15.4)	69 (16.2)	32 (10.4)	0.056
Extracardiac biopsy	714 (27.8)	399 (21.8)^*†^	140 (32.9)^‡^	175 (57.0)	<0.001
Nonbiopsy criteria	1469 (57.3)	1153 (62.9)^*†^	216 (50.8)^‡^	100 (32.6)	<0.001
Cardiac biomarkers					
NAC stage					<0.001
1	1267 (49.4)	866 (47.2)^†^	191 (44.9)^‡^	210 (68.4)	
2	888 (34.6)	664 (36.2)^†^	146 (34.4)^‡^	78 (25.4)	
3	411 (16.0)	304 (16.6)^†^	88 (20.7)^‡^	19 (6.2)	
NT‐proBNP, ng/L	2738 (1423–5109)	2907 (1499–5281)^†^	2850 (1548–5704)^‡^	1852 (626–3714)	<0.001
Troponin‐T, ng/L	58 (39–85)	58 (40–84)^*†^	73 (49–108)^‡^	40 (25–60)	<0.001
Troponin‐T >14 ng/L	2397 (98.2)	1742 (98.9)^†^	395 (99.0)^‡^	260 (92.5)	<0.001
Troponin‐T >56 ng/L	1262 (51.7)	915 (51.9)^*†^	266 (66.7)^‡^	82 (29.2)	<0.001
Full blood cell count					
Hemoglobin, g/L	136 (124–146)	137 (126–148)^*†^	129 (120–140)^‡^	134 (124–144)	<0.001
Anemia	990 (38.6)	716 (39.0)*	179 (42.1)^‡^	95 (30.9)	0.007
WBC count×10^9^/L	6.5 (5.4–7.8)	6.8 (5.8–6.9)^*†^	5.0 (4.2–6.1)^‡^	6.3 (5.2–7.5)	<0.001
Platelets×10^9^/L	201 (168–240)	202 (170–240)^*†^	190 (156–233)^‡^	208 (179–253)	<0.001
Low platelets	387 (15.1)	263 (14.4)*	91 (21.4)	33 (10.8)	<0.001
Serum biochemistry					
Sodium, mmol/L	141 (139–143)	141 (139–143)*	141 (139–143)	141 (139–143)	0.011
Hyponatremia	151 (5.9)	114 (6.2)	15 (3.5)	22 (7.2)	0.063
Potassium, mmol/L	4.5 (4.2–4.8)	4.5 (4.3–4.8)^*†^	4.5 (4.2–4.8)	4.4 (4.2–4.7)	<0.001
Urea, mmol/L	8.5 (6.5–11.2)	8.9 (6.9–11.7)^*†^	8.0 (6.2–11.0)^‡^	6.4 (5.2–8.3)	<0.001
High urea	1342 (52.3)	1061 (57.9)^*†^	201 (47.3)^‡^	80 (26.1)	<0.001
Creatinine, μmol/L	105 (87–128)	107 (90–131)^†^	110 (92–133)^‡^	81 (68–98)	<0.001
eGFR, mL/min per 1.73 m^2^	60 (47–74)	59 (46–72)^*†^	55 (43–67)^‡^	80 (64–90)	<0.001
eGFR <60 mL/min per 1.73 m^2^	1271 (49.5)	953 (52.0)^*†^	261 (61.4)^‡^	57 (18.6)	<0.001
Serum total bilirubin, μmol/L	13 (9–19)	12 (10–19)^†^	13 (8–20)^‡^	11 (8–15)	<0.001
Hyperbilirubinemia	459 (17.9)	337 (18.4)^†^	89 (20.9)^‡^	33 (10.7)	0.001
Alanine transaminase, IU/L	24 (19–32)	24 (19–31)*	27 (20–37)^‡^	24 (19–32)	<0.001
High alanine transaminase	192 (7.5)	99 (5.4)^*†^	60 (14.1)	33 (10.7)	<0.001
Aspartate aminotransferase, IU/L	29 (24–36)	29 (24–35)^*†^	32 (26–42)^‡^	26 (21–31)	<0.001
High aspartate aminotransferase	178 (6.9)	84 (4.6)*	75 (17.6)	19 (6.2)	<0.001
Alkaline phosphatase, IU/L	92 (72–124)	94 (74–143)^*†^	97 (76–134)^‡^	78 (62–103)	<0.001
High alkaline phosphatase	421 (16.4)	323 (17.6)^†^	80 (18.8)^‡^	18 (5.9)	<0.001
GGT, IU/L	69 (32–145)	69 (34–143)^*†^	104 (51–209)^‡^	33 (18–75)	<0.001
High GGT	1791 (69.8)	1308 (71.3)^*†^	350 (82.4)^‡^	133 (43.3)	<0.001
Total protein, g/L	71 (67–74)	71 (67–74)^*†^	73 (69–76)^‡^	68 (65–72)	<0.001
Albumin, g/L	44 (42–46)	44 (42–46)^*†^	42 (40–45)^‡^	43 (41–46)	<0.001
Hypoalbuminemia	38 (1.5)	16 (0.9)*	17 (4.0)^‡^	5 (1.6)	<0.001
CRP, mg/L	2 (1–5)	2 (1–5)^†^	3 (1–7)^‡^	1 (1–3)	<0.001
High CRP	700 (27.3)	509 (27.8)^†^	139 (32.7)^‡^	52 (16.9)	<0.001
Echocardiographic parameters					
IVSd, mm	16.8±2.5	16.9±2.5^†^	16.8±2.3^‡^	16.2±3.1	0.001
PWTd, mm	16.3±2.6	16.4±2.6^†^	16.5±2.4	15.9±3.1	0.009
RWT	0.78±0.18	0.77±0.17*	0.81±0.18	0.79±0.19	0.004
LVEF, %	48.2±10.6	48.9±10.2^*†^	43.7±11.0^‡^	50.8±10.9	<0.001
LVEF ≤40%	598 (23.3)	377 (20.6)*	169 (39.8)	52 (16.9)	<0.001
Stroke volume, mL	37.1±13.4	38.7±13.4*	30.8±11.8	36.8±12.7	<0.001
Longitudinal strain, %	−11.0±3.8	−11.1±3.7^*†^	−9.7±3.3^‡^	−11.9±4.3	<0.001
Average E/e’	16.8±6.5	16.4±6.3*	17.9±6.4	17.4±7.7	<0.001
Bone scintigraphy					
Degree of cardiac uptake					<0.001
Grade 0	3 (0.5)	0 (0.0)	0 (0.0)	3 (4.4)	
Grade 1	12 (0.5)	0 (0.0)*	0 (0.0)^‡^	12 (4.4)	
Grade 2	1880 (81.9)	1485 (89.4)^*†^	229 (63.1)	166 (61.0)	
Grade 3	401 (17.5)	176 (10.6)^*†^	134 (36.9)	91 (33.5)	

Data are given as mean±SD, number (percentage), and median (interquartile range). *P<0.05 for wtATTR vs p.(V142I) hATTR. ^†^ *P*<0.05 for wtATTR vs non‐p.(V142I) hATTR. ^‡^ *P*<0.05 for p.(V142I) hATTR vs non‐p.(V142I) hATTR. ATTR‐CA indicates transthyretin cardiac amyloidosis; CRP, C‐reactive protein; eGFR, estimated glomerular filtration rate; GGT, γ‐glutamyl transferase; hATTR‐CA, hereditary ATTR‐CA; IVSd, interventricular septal thickness in diastole; LVEF, left ventricular ejection fraction; NAC, National Amyloidosis Centre; NT‐pro‐BNP, N‐terminal pro‐B‐type natriuretic peptide; PWTd, posterior wall thickness in diastole; RWT, relative wall thickness; TIA, transient ischemic attack; WBC, white blood cell; and wtATTR‐CA, wild‐type ATTR‐CA.

### Blood Biomarkers and ATTR‐CA Genotype

**Figure 1:**
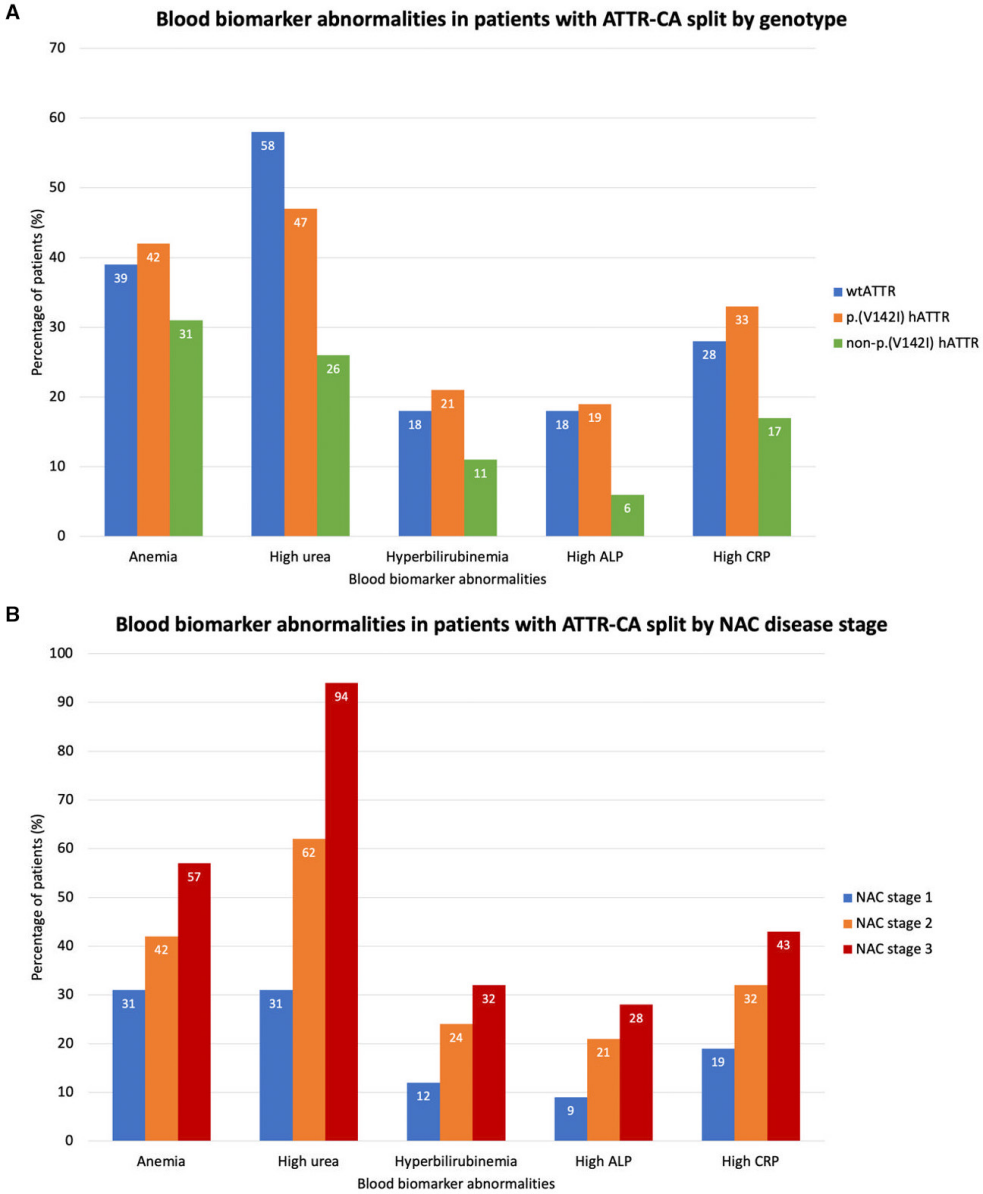
Prevalence of blood biomarker abnormalities. **A**, Blood biomarker abnormalities in patients with transthyretin cardiac amyloidosis (ATTR‐CA) split by genotype. **B**, Blood biomarker abnormalities in patients with ATTR‐CA split by National Amyloidosis Centre (NAC) disease stage. ALP indicates alkaline phosphatase; CRP, C‐reactive protein; hATTR‐CA, hereditary ATTR‐CA; and wtATTR‐CA, wild type ATTR‐CA.

At diagnosis, blood biomarkers differed significantly between genotypes. Patients with wtATTR‐CA and p.(V142I) hATTR‐CA had a significantly higher NT‐proBNP than patients with non‐p.(V142I) hATTR‐CA (Table 1, Figure 1A). This was reflected in the NAC staging system, with patients with p.(V142I) hATTR‐CA having the highest NAC stage, followed by those with wtATTR‐CA and non‐p.(V142I) hATTR‐CA. The overwhelming majority of patients had an elevated troponin level (98.2%), and patients with p.(V142I) hATTR‐CA had the highest median troponin‐T level, followed by those with wtATTR‐CA and non‐p.(V142I) hATTR‐CA.

Over one‐third (38.6%) of the patients were anemic, and anemia was most prevalent in p.(V142I) hATTR‐CA. Anemic patients with wtATTR‐CA had a significantly higher median (interquartile range) hemoglobin level than anemic patients with p.(V142I) hATTR‐CA (124 [115–129] versus 121 [109–128] ng/L; *P*<0.001); and the prevalence of severe anemia (hemoglobin <10.0 g/L) was higher in those with p.(V142I) hATTR‐CA than wtATTR‐CA and non‐p.(V142I) hATTR‐CA (22 [5.2%] versus 30 [1.6%] versus 2 [0.7%]; *P*<0.001).

Almost half (49.5%) had chronic kidney disease (CKD) stage 3 to 5, and 53.2% had a high urea level at diagnosis. CKD stage 3 to 5 was most prevalent in p.(V142I) hATTR‐CA, whereas a high urea was most prevalent in wtATTR‐CA.

Elevated cholestatic liver enzymes were also common (1812 [70.6%] had either an elevated γ‐glutamyl transferase or alkaline phosphatase [ALP]), with 69.8% having an increased γ‐glutamyl transferase and 16.4% having an increased ALP. This was more prevalent than transaminitis (elevated alanine transaminase or aspartate aminotransferase), which was present in only 271 (10.5%) patients. Those with wtATTR‐CA and p.(V142I) hATTR‐CA had significantly higher liver enzyme levels than those with non‐p.(V142I) hATTR‐CA. Synthetic liver function was also affected, with hyperbilirubinemia in 17.9% of patients and hypalbuminaemia in 1.5%, both of which were most prevalent in patients with p.(V142I) hATTR‐CA. Over one‐quarter (27.3%) had an increased serum CRP (C‐reactive protein) at diagnosis. Those with wtATTR‐CA and p.(V142I) hATTR‐CA had a significantly higher CRP, and more commonly had an elevated CRP than those with non‐p.(V142I) hATTR‐CA (Table 1, Table S2, Figure 1A).

### Blood Biomarkers and Disease Severity

Patients with a higher NAC disease stage at diagnosis had a higher prevalence of anemia, hyponatremia, high urea, deranged liver function test results, and an elevated CRP and troponin‐T. Most significant differences observed were between patients with stage 1 (mild) and patients with NAC stage 2 (moderate) or 3 (severe) disease. However, there were some significant differences between patients with NAC stage 2 and stage 3 disease. Over half of the patients diagnosed with NAC stage 3 disease were anemic (56.7%), the overwhelming majority were uremic (94.4%), nearly one‐third had hyperbilirubinemia (32.1%), over one‐fifth had an elevated ALP (28.2%), and nearly half had an elevated CRP (Table 2, Table S3, Figure 1B).

**Table 2:** Blood Biomarkers Split by NAC Disease Stage

Biomarker	NAC stage 1 (n=1267, 49.4%)	NAC stage 2 (n=888, 34.6%)	NAC stage 3 (n=411, 16.0%)	*P* value
Full blood cell count				
Hemoglobin, g/L	138 (128–148)^*†^	135 (124–146)^‡^	126 (115–139)	<0.001
Anemia	390 (30.8)^*†^	367 (41.3)^‡^	233 (56.7)	<0.001
WBC count×10^9^/L	6.5 (5.4–7.8)	6.6 (5.4–7.9)	6.4 (5.2–7.8)	0.396
Platelets×10^9^/L	206 (175–247)^*†^	197 (162–238)	189 (156–232)	<0.001
Low platelets	137 (10.8)^*†^	162 (18.3)	88 (21.5)	<0.001
Serum biochemistry				
Sodium, mmol/L	141 (139–143)^*†^	141 (138–143)	140 (138–142)	<0.001
Hyponatremia	54 (4.3)^*†^	67 (7.5)	30 (7.3)	0.003
Potassium, mmol/L	4.5 (4.3–4.8)^*†^	4.5 (4.2–4.8)	4.6 (4.3–5.0)	<0.001
Urea, mmol/L	7.1 (5.8–8.7)^*†^	9.3 (7.3–11.6)^‡^	14.5 (11.5–19.2)	<0.001
High urea	397 (31.3)^*†^	557 (62.7)^‡^	388 (94.4)	<0.001
Creatinine, μmol/L	93 (79–107)^*†^	110 (94–126)^‡^	160 (144–184)	<0.001
Serum total bilirubin, μmol/L	11 (8–11)^*†^	15 (10–21)	14 (10–21)	<0.001
Hyperbilirubinemia	155 (12.2)^*†^	209 (23.5)	95 (32.1)	<0.001
Alanine transaminase, IU/L	25 (19–33)^†^	25 (20–34)^‡^	23 (18–30)	<0.001
High alanine transaminase	90 (7.1)	71 (8.0)	31 (7.5)	0.740
Aspartate aminotransferase, IU/L	28 (23–34)^*†^	30 (25–37)	29 (24–37)	<0.001
High aspartate aminotransferase	67 (5.3)^†^	66 (7.4)	45 (10.9)	<0.001
Alkaline phosphatase, IU/L	83 (67–106)^*†^	102 (77–137)^‡^	114 (87–155)	<0.001
High alkaline phosphatase	115 (9.1)^*†^	190 (21.4)^‡^	116 (28.2)	<0.001
GGT, IU/L	48 (26–107)^*†^	88 (44–184)^‡^	108 (55–210)	<0.001
High GGT	738 (58.2)^*†^	707 (79.6)^‡^	346 (84.2)	<0.001
Albumin, g/L	44 (42–46)^*†^	43 (41–45)	43 (41–45)	<0.001
Hypoalbuminemia	9 (0.7)^*†^	17 (1.9)	12 (2.9)	0.002
CRP, mg/L	2 (1–4)^*†^	3 (1–6)^‡^	4 (2–8)	<0.001
High CRP	242 (19.1)^*†^	281 (31.6)^‡^	177 (43.1)	<0.001
NT‐proBNP, ng/L	1468 (846–2155)^*†^	4533 (3374–6494)^‡^	6952 (4703–10 981)	<0.001
Troponin‐T, ng/L	43 (29–60)^*†^	69 (51–94)^‡^	104 (74–146)	<0.001
Troponin‐T >14 ng/L	1163 (96.5)^*†^	841 (99.6)	393 (100.0)	<0.001
Troponin‐T >56 ng/L	355 (29.5)^*†^	565 (66.9)^‡^	342 (87.0)	<0.001
Echocardiographic parameters				
IVSd, mm	16.3±2.6^*†^	17.3±2.4	17.2±2.4	<0.001
PWTd, mm	15.8±2.6^*†^	16.9±2.4	16.8±2.6	<0.001
RWT	0.75±0.17^*†^	0.80±0.18	0.81±0.19	<0.001
LVEF, %	51.0±9.5^*†^	45.8±10.7	44.9±11.3	<0.001
LVEF ≤40%	185 (14.6)^*†^	270 (30.4)	143 (34.8)	<0.001
Stroke volume, mL	39.7±13.7^*†^	35.5±12.5	33.0±12.9	<0.001
Longitudinal strain, %	−12.2±3.8^*†^	−9.8±3.2	−9.5±3.3	<0.001
Average E/e’	15.6±5.9^*†^	17.9±6.9	18.1±6.9	<0.001

Data are given as mean±SD, number (percentage), and median (interquartile range). *P* values for pairwise comparison: α=*P*<0.01 for NAC stage 1 vs NAC stage 2, β=*P*<0.01 NAC stage 1 vs NAC stage 3, γ=*P*<0.01 NAC stage 2 vs NAC stage 3. CRP indicates C‐reactive protein; GGT, γ‐glutamyl transferase; IVSd, interventricular septal thickness in diastole; LVEF, left ventricular ejection fraction; NAC, National Amyloidosis Centre; NT‐pro‐BNP, N‐terminal pro‐B‐type natriuretic peptide; PWTd, posterior wall thickness in diastole; RWT, relative wall thickness; and WBC, white blood cell.

New York Heart Association (NYHA) class was available in 2409 (93.9%) patients, most of whom were in NYHA class II (n=1531, 63.6%). Similar trends were observed when patients were split by NYHA class. Patients with a higher NYHA class at diagnosis had a higher prevalence of anemia, high urea, deranged liver function test results, and elevated CRP and troponin‐T. Most significant differences were observed between patients with NYHA functional class I to II and NYHA class III to IV. However, there were some significant differences between patients with NYHA functional class III and NYHA class IV. Patients with NYHA functional class IV had a lower hemoglobin, higher aspartate aminotransferase, higher prevalence of hypoalbuminemia, and higher CRP (Table S4).

When divided by left ventricular ejection fraction (LVEF), patients with an LVEF ≤40% had a higher prevalence of high urea, CKD stage 3 to 5, hyperbilirubinemia, deranged liver function test results, and elevated CRP and troponin‐T, than patients with a LVEF >40% (Table S5).

Across the overall population, stroke volume had a weak negative correlation with serum urea, bilirubin, aspartate aminotransferase, ALP, γ‐glutamyl transferase, CRP, and troponin‐T, and a weak positive correlation with serum albumin (Table S6). Multivariable linear regression demonstrated that stroke volume had an independent positive relationship with serum albumin, and an independent negative relationship with serum bilirubin (Table S7).

### Survival

At median follow up of 37.1 months (interquartile range, 16.4–59.2 months), 966 (37.6%) patients had died, and the death rate was 12.7 deaths per 100 person‐years (95% CI, 12.0–13.6). There were 638 (34.8%) deaths in the wtATTR‐CA group, with a death rate of 11.8 deaths per 100 person‐years (95% CI, 10.9–12.8), 228 (53.6%) in the p.(V142I) hATTR‐CA group, with a death rate of 19.7 deaths per 100 person‐years (95% CI, 17.2–22.2), and 100 (32.6%) in the non‐p.(V142I) hATTR‐CA group, with a death rate of 9.7 deaths per 100 person‐years (95% CI, 7.9–11.8). Blood biomarkers first were explored in a univariable Cox regression analysis as continuous variables, z‐score standardized variables, and dichotomous variables, and all except for alanine transaminase were associated with mortality (Table S8). Following z‐score standardization, blood biomarkers were also investigated in a multivariable Cox regression analysis, and hemoglobin, serum sodium, urea, bilirubin, albumin, and troponin all remained independently associated with mortality (Table S9).

**Figure 2:**
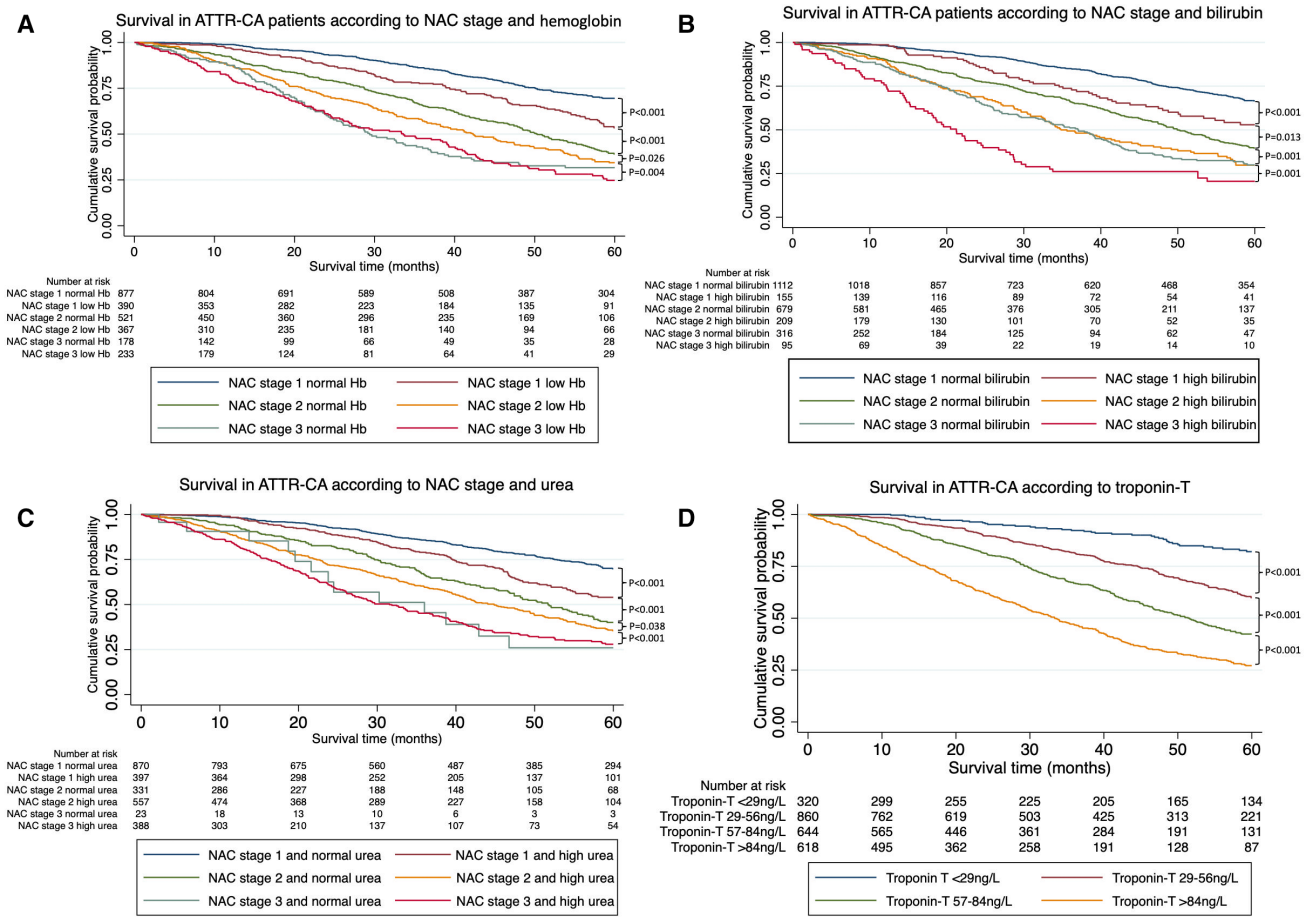
Blood biomarker abnormalities and survival. **A**, Kaplan‐Meier curve demonstrating the effect of National Amyloidosis Centre (NAC) disease stage and anemia on survival. **B**, Kaplan‐Meier curve demonstrating the effect of NAC disease stage and hyperbilirubinemia on survival. **C**, Kaplan‐Meier curve demonstrating the effect of NAC disease stage and high urea on survival. **D**, Kaplan‐Meier curves demonstrating the effect of troponin on survival. Troponin‐T 29 to 56 ng/L vs troponin‐T <29 ng/L: hazard ratio (HR), 2.59 (95% CI, 1.85–3.63), *P*<0.001; troponin‐T 57 to 84 ng/L vs troponin‐T 29 to 56 ng/L: HR, 1.79 (95% CI, 1.50–2.13), *P*<0.001; troponin‐T >84 ng/L vs troponin‐T 57 to 84 ng/L: HR, 1.77 (95% CI, 1.51–2.07), *P*<0.001. ATTR‐CA indicates transthyretin cardiac amyloidosis; and Hb, hemoglobin.

The association between blood biomarkers as dichotomous variables and mortality within individual NAC stages was further investigated. Within all 3 NAC disease subgroups, hyperbilirubinemia, hypoalbuminemia, high ALP, high CRP, hyponatremia, and troponin‐T >56 ng/L were associated with mortality. However, anemia and high urea were associated with mortality in the subgroups with NAC stage 1 and NAC stage 2 disease, but not in those with NAC stage 3 disease. The likelihood ratio test demonstrated that each blood biomarker added a significant contribution to the NAC disease stage (anemia: χ^2^=16.92, *P*<0.001; hyperbilirubinemia: χ^2^=28.78, *P*<0.001; high urea: χ^2^=18.29, *P*<0.001; hypoalbuminemia: χ^2^=16.12, *P*<0.001; high ALP: χ^2^=26.81, *P*<0.001; high CRP: χ^2^=19.93, *P*<0.001; hyponatremia: χ^2^=15.62, *P*<0.001; troponin‐T >56 ng/L: χ^2^=88.67, *P*<0.001) (Figure 2).

**Figure 3:**
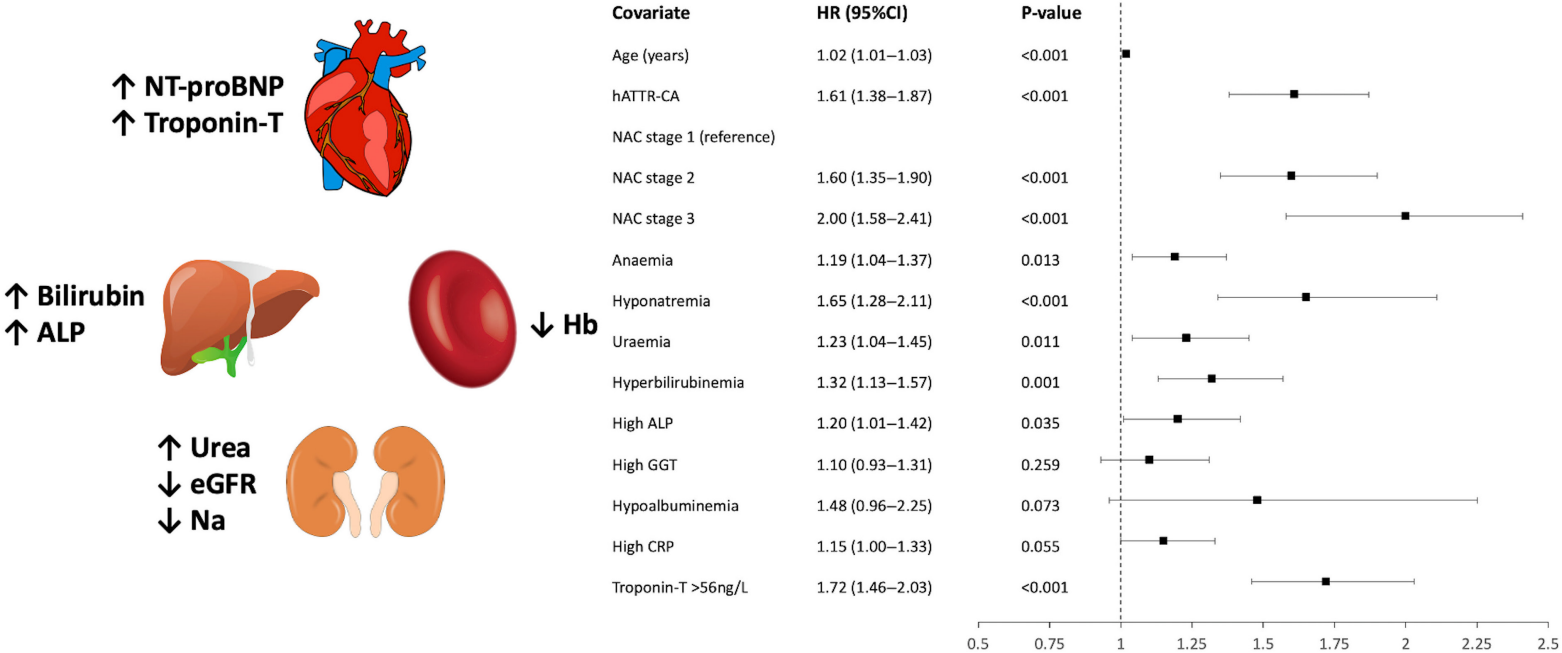
The association of blood biomarker abnormalities with mortality in transthyretin cardiac amyloidosis (ATTR‐CA). Multivariable analysis demonstrating the cardiac and noncardiac blood biomarkers that are independently associated with mortality. ALP indicates alkaline phosphatase; CRP, C‐reactive protein; eGFR, estimated glomerular filtration rate; GGT, γ‐glutamyl transferase; hATTR‐CA, hereditary ATTR‐CA; Hb, hemoglobin; HR, hazard ratio; Na, sodium; NAC, National Amyloidosis Centre; and NT‐proBNP, N‐terminal pro‐B‐type natriuretic peptide.

Blood biomarkers selected a priori based on clinical relevance were explored in a multivariable model that adjusted for known predictors of prognosis (age, NAC stage, and wtATTR‐CA or hATTR‐CA). This model confirmed that anemia, hyponatremia, high urea, hyperbilirubinemia, elevated ALP, and troponin‐T >56 ng/L remained independent predictors of mortality; and all except for elevated ALP remained independent predictors when patients were censored at the start date of disease‐modifying therapy and enrollment into clinical trials (Table 3, Figure 3). Multivariable Cox regression models, adjusting for furosemide equivalent dose and stroke volume, confirmed that anemia, hyponatremia, high urea, hyperbilirubinemia, and troponin‐T >56 ng/L all remained independently associated with mortality (Table S10).

**Table 3:** Multivariable Cox Proportional Hazards Regression Analysis of Risk of Death in the Overall Population With ATTR‐CA

Multivariable Cox regression analysis in the overall population					
Patients not censored for the start date of clinical trials and disease‐modifying therapy			Patients censored for the start date of clinical trials and disease‐modifying therapy		
Variable	HR (95% CI)	*P* value	Variable	HR (95% CI)	*P* value
Age, y	1.02 (1.01–1.03)	<0.001	Age, y	1.02 (1.01–1.03)	<0.001
hATTR‐CA	1.61 (1.38–1.87)	<0.001	hATTR‐CA	1.59 (1.35–1.87)	<0.001
NAC stage 1	Reference		NAC stage 1	Reference	
NAC stage 2	1.60 (1.35–1.90)	<0.001	NAC stage 2	1.61 (1.34–1.92)	<0.001
NAC stage 3	2.00 (1.58–2.41)	<0.001	NAC stage 3	2.02 (1.62–2.51)	<0.001
Anemia	1.19 (1.04–1.37)	0.013	Anemia	1.18 (1.02–1.36)	0.024
Hyponatremia	1.65 (1.28–2.11)	<0.001	Hyponatremia	1.69 (1.30–2.19)	<0.001
High urea	1.23 (1.04–1.45)	0.011	High urea	1.23 (1.04–1.46)	0.018
Hyperbilirubinemia	1.32 (1.13–1.57)	0.001	Hyperbilirubinemia	1.30 (1.10–1.54)	0.002
High ALP	1.20 (1.01–1.42)	0.035	High ALP	1.19 (1.00–1.41)	0.054
High GGT	1.10 (0.93–1.31)	0.259	High GGT	1.09 (0.91–1.30)	0.351
Hypoalbuminemia	1.48 (0.96–2.25)	0.073	Hypoalbuminemia	1.50 (0.98–2.30)	0.061
High CRP	1.15 (1.00–1.33)	0.055	High CRP	1.14 (0.98–1.32)	0.094
Troponin‐T >56 ng/L	1.72 (1.46–2.03)	<0.001	Troponin‐T >56 ng/L	1.66 (1.39–1.97)	<0.001
Harrell's C statistic	0.722 (0.705–0.739)	<0.001	Harrell's C statistic	0.719 (0.701–0.737)	<0.001

ALP indicates alkaline phosphatase; ATTR‐CA, transthyretin cardiac amyloidosis; CRP, C‐reactive protein; GGT, γ‐glutamyl transferase; hATTR‐CA, hereditary ATTR‐CA; HR, hazard ratio; and NAC, National Amyloidosis Centre.

The multivariable model was repeated in the prespecified subgroups of patients with an LVEF ≤40% and patients with an LVEF >40%. In patients with an LVEF ≤40%, anemia, hyponatremia, high ALP, and troponin‐T >56 ng/L were all independently associated with mortality; whereas in patients with an LVEF >40%, hyponatremia, high urea, hyperbilirubinemia, and troponin‐T >56 ng/L were all independently associated with mortality (Table S11).

A similar multivariable model was used in patients with wtATTR‐CA and p.(V142I) hATTR‐CA separately. The model was not applied to non‐p.(V142I) hATTR‐CA because of a low number of events. In wtATTR‐CA, hyponatremia, high urea, hyperbilirubinemia, hypoalbuminemia, and troponin‐T >56 ng/L remained independently associated with mortality; whereas in p.(V142I) hATTR‐CA, only anemia and troponin‐T >56 ng/L remained independently associated with mortality (Table 4).

**Table 4:** Multivariable Cox Proportional Hazards Regression Analysis of Risk of Death in the Overall Population With ATTR‐CA, and in the Prespecified Subgroups of Patients With wtATTR‐CA and p.(V142I) hATTR‐CA

Multivariable Cox regression analysis for patients with wtATTR‐CA					
Patients not censored for the start date of clinical trials and disease‐modifying therapy			Patients censored for the start date of clinical trials and disease‐modifying therapy		
Variable	HR (95% CI)	*P* value	Variable	HR (95% CI)	*P* value
Age, y	1.03 (1.01–1.04)	<0.001	Age, y	1.02 (1.01–1.04)	0.001
NAC stage 1	Reference		NAC stage 1	Reference	
NAC stage 2	1.56 (1.26–1.94)	<0.001	NAC stage 2	1.55 (1.23–1.95)	<0.001
NAC stage 3	2.08 (1.60–2.70)	<0.001	NAC stage 3	2.13 (1.63–2.79)	<0.001
Anemia	1.05 (0.88–1.24)	0.607	Anemia	1.04 (0.87–1.23)	0.693
Hyponatremia	1.99 (1.51–2.63)	<0.001	Hyponatremia	2.01 (1.51–2.69)	<0.001
High urea	1.44 (1.17–1.78)	0.001	High urea	1.48 (1.19–1.85)	<0.001
Hyperbilirubinemia	1.58 (1.31–1.92)	<0.001	Hyperbilirubinemia	1.55 (1.26–1.90)	<0.001
High ALP	1.17 (0.96–1.44)	0.124	High ALP	1.19 (0.97–1.48)	0.095
High GGT	1.04 (0.84–1.28)	0.729	High GGT	1.02 (0.82–1.26)	0.095
Hypoalbuminemia	2.12 (1.17–3.83)	0.013	Hypoalbuminemia	2.13 (1.18–3.87)	0.013
High CRP	1.19 (1.00–1.42)	0.052	High CRP	1.14 (0.95–1.37)	0.153
Troponin‐T >56 ng/L	1.83 (1.48–2.26)	<0.001	Troponin‐T >56 ng/L	1.66 (1.33–2.06)	<0.001
Harrell's C statistic	0.736 (0.716–0.756)	<0.001	Harrell's C statistic	0.730 (0.709–0.752)	<0.001

Multivariable Cox regression analysis for patients with p.(V142I) hATTR‐CA					
Patients not censored for the start date of clinical trials and disease‐modifying therapy			Patients censored for the start date of clinical trials and disease‐modifying therapy		
Variable	HR (95% CI)	*P* value	Variable	HR (95% CI)	*P* value
Age, y	1.03 (1.01–1.05)	0.008	Age, y	1.02 (1.00–1.05)	0.101
NAC stage 1	Reference		NAC stage 1	Reference	
NAC stage 2	1.40 (0.99–1.97)	0.053	NAC stage 2	1.54 (1.09–2.18)	0.015
NAC stage 3	1.63 (1.08–2.48)	0.021	NAC stage 3	1.79 (1.17–2.73)	0.007
Anemia	1.58 (1.17–2.12)	0.003	Anemia	1.52 (1.12–2.07)	0.007
Hyponatremia	1.25 (0.58–2.71)	0.567	Hyponatremia	1.52 (0.69–3.33)	0.295
High urea	1.14 (0.82–1.58)	0.442	High urea	1.05 (0.75–1.47)	0.779
Hyperbilirubinemia	0.94 (0.65–1.36)	0.752	Hyperbilirubinemia	0.89 (0.61–1.30)	0.551
High ALP	1.23 (0.85–1.78)	0.275	High ALP	1.16 (0.79–1.68)	0.448
High GGT	1.44 (0.92–2.25)	0.112	High GGT	1.39 (0.88–2.19)	0.162
Hypoalbuminemia	1.15 (0.59–2.22)	0.275	Hypoalbuminemia	1.14 (0.59–2.19)	0.705
High CRP	1.12 (0.83–1.51)	0.453	High CRP	1.21 (0.90–1.64)	0.210
Troponin‐T >56 ng/L	1.63 (1.12–2.36)	0.010	Troponin‐T >56 ng/L	1.70 (1.16–2.50)	0.007
Harrell's C statistic	0.690 (0.654–0.726)	<0.001	Harrell's C statistic	0.691 (0.654–0.727)	<0.001

ALP indicates alkaline phosphatase; ATTR‐CA, transthyretin cardiac amyloidosis; CRP, C‐reactive protein; GGT, γ‐glutamyl transferase; hATTR‐CA, hereditary ATTR‐CA; HR, hazard ratio; NAC, National Amyloidosis Centre; and wtATTR‐CA, wild type ATTR‐CA.

Troponin‐T >56 ng/L was consistently an independent predictor of mortality throughout all the multivariable models. Patients with ATTR‐CA could be further divided into prognostic categories based on troponin‐T, and with every increase of 28 ng/L, there was a significantly increased risk of mortality (Figure 2).

## Discussion

This study comprehensively evaluated the multifaceted nature of ATTR‐CA using a wide range of cardiac and noncardiac blood biomarker profiles, and assessed the association between blood biomarkers and mortality. Our study demonstrated that: (1) ATTR‐CA is a complex and multisystemic disease process that commonly presents with a range of cardiac and noncardiac blood biomarker abnormalities; (2) blood biomarker abnormalities are most prevalent in patients with p.(V142I) hATTR‐CA, and become more prevalent as the cardiac disease severity increases; and (3) noncardiac blood biomarkers are associated with mortality, anemia, hyponatremia, high urea, hyperbilirubinemia, and increased ALP, remaining independently associated with mortality after adjusting for troponin‐T, NT‐proBNP, and estimated glomerular filtration rate. This highlights the importance of multisystemic disease in ATTR‐CA, whereby multiple pathways involving different organs and systems, beyond cardiac infiltration, contribute to disease progression and prognosis (Figure 3).

HF is increasingly recognized as a multifaceted disease process. The underlying pathophysiology is more complex than can be explained by the simple concept of pump failure. Recent studies have demonstrated that multiple hematological, biochemical, and inflammatory homeostatic pathways are influenced by the deterioration of cardiac function.^6–^ ^8^, ^10^ Anemia, congestive hepatopathy, and systemic inflammation have emerged as common comorbidities that independently predict outcomes in patients with HF.^6^, ^7^, ^8^ The same is true for the population with ATTR‐CA, with over one‐third of patients being anemic, over two‐thirds having elevated cholestatic enzymes, and over one‐quarter having an elevated CRP at diagnosis.

The pattern and prevalence of biomarker abnormalities varies between genotypes. Patients with p.(V142I) hATTR‐CA had a higher prevalence of anemia, deranged liver function, and a higher troponin than those with wtATTR‐CA and non‐p.(V142I) hATTR. Patients with p.(V142I) hATTR‐CA had more advanced cardiac disease at diagnosis, and the prevalence of biomarker abnormalities increased as the severity of cardiac disease increased, as evidenced by NAC disease stage, NYHA class, and LVEF. Therefore, the higher prevalence of biomarker abnormalities in p.(V142I) hATTR‐CA likely reflects more advanced cardiac disease at diagnosis. However, these observations could also relate to different demographics between genotypes. The overwhelming majority of patients with p.(V142I) hATTR‐CA were Afro‐Caribbean, and a greater proportion were women, compared with those with wtATTR‐CA, both of which have previously been associated with anemia in patients with HF.^11^, ^12^ This may have been compounded by the greater prevalence of CKD in p.(V142I) hATTR‐CA. Interestingly, patients with p.(V142I) hATTR‐CA had a higher troponin‐T, more severe degree of systolic and diastolic dysfunction, but similar NT‐proBNP to wtATTR‐CA. This could reflect a greater degree of ongoing myocyte damage as a consequence of intrinsic differences in disease biology, with p.(V142I) hATTR‐CA being associated with a more rapidly progressive disease. However, the relatively lower NT‐proBNP elevation in p.(V142I) hATTR‐CA could also reflect racial differences in natriuretic peptide synthesis and excretion, with Afro‐Caribbean patients being known to have lower NT‐proBNP levels.^13^ The p.(V142I) genotype was also associated with increased left ventricular filling pressures, and is known to cause a disproportionally greater degree of right ventricular systolic dysfunction and tricuspid regurgitation.^14^ The resulting increased hepatic congestion is likely to account for the increased prevalence of cholestasis in patients with p.(V142I) hATTR‐CA. Hypoalbuminemia has been also associated with hemodilution, inflammation, malnutrition, and cachexia in HF.^15^ Within the population with hATTR‐CA, albumin levels are influenced by the presence of concomitant polyneuropathy, and more specifically autonomic neuropathy, which results in impaired gastrointestinal motility and malabsorption.^16^ This is reflected in our population, with patients with hATTR‐CA having a lower serum total protein and lower albumin than those with wtATTR‐CA, and further illustrates the complexity of this multisystemic disease process.

In the current study, which represents the largest study of blood biomarkers in ATTR‐CA to date, anemia, hyponatremia, high urea, hyperbilirubinemia, and troponin‐T >56 ng/L were all independently associated with mortality, after adjusting for NT‐proBNP and estimated glomerular filtration rate. Throughout the spectrum of genotypes and LVEF, troponin‐T >56 ng/L remained independently associated with mortality. This is likely to reflect the importance of ongoing myocyte death resulting from constant amyloid fibril accumulation, and demonstrates the importance of troponin‐T measurements in stratifying prognosis and identifying high‐risk patients.^17^ However, the prognostic impact of noncardiac biomarkers varied across the spectrum of genotypes and LVEF. Anemia was associated with mortality in p.(V142I) hATTR‐CA across the spectrum of LVEF and the subgroup of patients with an LVEF ≤40% from the overall population; but not in the subgroups of wtATTR‐CA and patients with an LVEF >40%. The difference in prognostic impact is possibly attributable to differences in genotype characteristics and disease severity. First, p.(V142I) hATTR‐CA was not only more commonly associated with anemia (likely because of the higher prevalence of female and Afro‐Caribbean patients) but was also associated with more severe anemia than wtATTR‐CA (possibly because of the higher prevalence of CKD). Second, p.(V142I) hATTR‐CA accounted for a greater proportion of patients with an LVEF ≤40% than patients with an LVEF >40%. In patients with ATTR‐CA, anemia was only independently associated with mortality in the subgroup with a LVEF ≤40%, and this is consistent with previous studies of patients with HF that demonstrated a greater prognostic importance of anemia in patients with a reduced LVEF.^18^ Iron‐deficiency anemia not only represents a common comorbidity in HF, but has emerged as a modifiable treatment target, with restoration of iron levels resulting in improved functional capacity and prognosis in the Ferinject Assessment in Patients with Iron Deficiency and Chronic Heart Failure and Effectiveness of Intravenous Iron Treatment versus Standard Care in Patients with Heart Failure and Iron Deficiency trials.^19^, ^20^ Currently, tafamidis (a highly specific transthyretin stabilizer) is the only treatment proven to improve prognosis in ATTR‐CA, but despite efficacy, the associated high cost has resulted in restricted use.^21^, ^22^ Analysis of HF medications in ATTR‐CA demonstrated that neurohormonal modulation through mineralocorticoid receptor antagonists and low‐dose β‐blockers in patients with an LVEF ≤40% was associated with improved survival; and suggests ATTR‐CA may share similar pathophysiological mechanisms to HF of different causes.^23^ Considering these findings alongside data demonstrating the independent association between anemia and mortality, it is plausible that correction of anemia in ATTR‐CA could improve outcomes, especially in patients with p.(V142I) hATTR‐CA or patients with an LVEF ≤40%. However, the correction of anemia as a possible modifiable risk factor has not been featured in the recent consensus documents by the European Society of Cardiology and American College of Cardiology for the treatment ATTR‐CA.^24^, ^25^ Although the analysis reported here has limitations, the observations do raise the question as to whether there could be some benefit from correcting anemia, and therefore this should be explored as a potential treatment option in prospective randomized controlled clinical trials.

### Limitations

As a retrospective study, we have been able to demonstrate the association between biomarkers and mortality, but are unable to prove causality. All‐cause mortality was obtained from a national database, but data on cardiovascular mortality were not available in all patients. Differences between genotypes could reflect a different propensity to seek medical attention and different referral practices across populations. However, this is likely to be partially mitigated by the free nature of the National Health Service at the point of care, and hence, the decision to seek medical attention is less likely to be influenced by socioeconomic status.

## Conclusions

In this large cohort of patients with ATTR‐CA, both cardiac and noncardiac blood biomarker abnormalities were common and were more prevalent in patients with p.(V142I) hATTR‐CA and advanced cardiac disease. The wide range of abnormalities reflects the complex and multifaceted nature of ATTR‐CA and highlights the importance of multisystemic disease, whereby multiple pathways involving different organs and systems, beyond cardiac infiltration, contribute to disease progression and prognosis. Whether treatment aimed at restoring hemoglobin levels in this population would potentially reduce the risk of mortality remains unknown. Therefore, clinical trials are needed to investigate whether biomarker abnormalities represent modifiable risk factors that if specifically targeted could improve outcomes.

## Sources of Funding

Dr Fontana is supported by a British Heart Foundation Intermediate Clinical Research Fellowship (FS/18/21/33447). Dr Knight is supported by a British Heart Foundation Clinical Research Leave Fellowship (FS/CRLF/20/23004).

## Disclosures

Dr Fontana has consulting income from Intellia, Novo Nordisk, Pfizer, Eidos, Prothena, Alnylam, Alexion, Janssen, and Ionis. Dr Gillmore has consulting income from Ionis, Alexion, Eidos, Intellia, Alnylam, and Pfizer. Dr Wechalekar has consulting income from Alexia, AstraZeneca, Janssen, Attralus, and Prothena. Dr Hawkins has consulting income from Alnylam. The remaining authors have no disclosures to report.

## Supporting information

Tables S1–S11.

## References

[1] Ioannou A , Patel RK , Razvi Y , Porcari A , Sinagra G , Venneri L , Bandera F , Masi A , Williams GE , O'Beara S , et al. Impact of earlier diagnosis in cardiac ATTR amyloidosis over the course of 20 years. Circulation. 2022;146:1657–1670. doi: 10.1161/CIRCULATIONAHA.122.060852 36325894 PMC9698091

[2] Porcari A , Razvi Y , Masi A , Patel R , Ioannou A , Rauf MU , Hutt DF , Rowczenio D , Gilbertson J , Martinez‐Naharro A , et al. Prevalence, characteristics and outcomes of older patients with hereditary versus wild‐type transthyretin amyloid cardiomyopathy. Eur J Heart Fail. 2023;25:515–524. doi: 10.1002/ejhf.2776 36644836

[3] Patel RK , Ioannou A , Razvi Y , Chacko L , Venneri L , Bandera F , Knight D , Kotecha T , Martinez‐Naharro A , Masi A , et al. Sex differences among patients with transthyretin amyloid cardiomyopathy–from diagnosis to prognosis. Eur J Heart Fail. 2022;24:2355–2363. doi: 10.1002/ejhf.2646 36575133 PMC10087683

[4] Ioannou A , Patel RK , Razvi Y , Porcari A , Knight D , Martinez‐Naharro A , Kotecha T , Venneri L , Chacko L , Brown J , et al. Multi‐imaging characterization of cardiac phenotype in different types of amyloidosis. JACC Cardiovasc Imaging. 2022;16:464–477. doi: 10.1016/j.jcmg.2022.07.008 36648052

[5] Gillmore JD , Damy T , Fontana M , Hutchinson M , Lachmann HJ , Martinez‐Naharro A , Quarta CC , Rezk T , Whelan CJ , Gonzalez‐Lopez E , et al. A new staging system for cardiac transthyretin amyloidosis. Eur Heart J. 2018;39:2799–2806. doi: 10.1093/eurheartj/ehx589 29048471

[6] Allen LA , Felker GM , Pocock S , McMurray JJ , Pfeffer MA , Swedberg K , Wang D , Yusuf S , Michelson EL , Granger CB , et al. Liver function abnormalities and outcome in patients with chronic heart failure: data from the Candesartan in Heart Failure: Assessment of Reduction in Mortality and Morbidity (CHARM) program. Eur J Heart Fail. 2009;11:170–177. doi: 10.1093/eurjhf/hfn031 19168515 PMC2639422

[7] Anand IS , Gupta P . Anemia and iron deficiency in heart failure. Circulation. 2018;138:80–98. doi: 10.1161/CIRCULATIONAHA.118.030099 29967232

[8] Adamo L , Rocha‐Resende C , Prabhu SD , Mann DL . Reappraising the role of inflammation in heart failure. Nat Rev Cardiol. 2020;17:269–285. doi: 10.1038/s41569-019-0315-x 31969688

[9] Liu X . Classification accuracy and cut point selection. Stat Med. 2012;31:2676–2686. doi: 10.1002/sim.4509 22307964

[10] Ioannou A , Rauf MU , Patel RK , Razvi Y , Porcari A , Martinez‐Naharro A , Venneri L , Bandera F , Virsinskaite R , Kotecha T , et al. Albuminuria in transthyretin cardiac amyloidosis: prevalence, progression and prognostic importance [published online November 23, 2023]. Eur J Heart Fail. doi: 10.1002/ejhf.3094 37997196

[11] Savitz ST , Leong T , Sung SH , Lee K , Rana JS , Tabada G , Go AS . Contemporary reevaluation of race and ethnicity with outcomes in heart failure. J Am Heart Assoc. 2021;10:1–17. doi: 10.1161/JAHA.120.016601 PMC7955425 33474975

[12] Go AS , Yang J , Ackerson LM , Lepper K , Robbins S , Massie BM , Shlipak MG . Hemoglobin level, chronic kidney disease, and the risks of death and hospitalization in adults with chronic heart failure. Circulation. 2006;113:2713–2723. doi: 10.1161/CIRCULATIONAHA.105.577577 16754803

[13] Bajaj NS , Gutiérrez OM , Arora G , Judd SE , Patel N , Bennett A , Prabhu SD , Howard G , Howard VJ , Cushman M , et al. Racial differences in plasma levels of N‐terminal pro–B‐type natriuretic peptide and outcomes: the Reasons for Geographic and Racial Differences in Stroke (REGARDS) study. JAMA Cardiol. 2018;3:11–17. doi: 10.1001/jamacardio.2017.4207 29167879 PMC5833525

[14] Razvi Y , Ioannou A , Patel RK , Chacko L , Karia N , Riefolo M , Porcari A , Rauf MU , Starr N , Ganesananthan S , et al. Deep phenotyping of p.(V142I)‐associated variant ATTR amyloid cardiomyopathy: distinct from wild‐type ATTR amyloidosis? [published online November 13, 2023]. Eur J Heart Fail. doi: 10.1002/ejhf.3088 37953725

[15] Liu M , Chan CP , Yan BP , Zhang Q , Lam YY , Li RJ , Sanderson JE , Coats AJ , Sun JP , Yip GW , et al. Albumin levels predict survival in patients with heart failure and preserved ejection fraction. Eur J Heart Fail. 2012;14:39–44. doi: 10.1093/eurjhf/hfr154 22158777

[16] Adams D , Algalarrondo V , Polydefkis M , Sarswat N , Slama MS , Nativi‐Nicolau J . Expert opinion on monitoring symptomatic hereditary transthyretin‐mediated amyloidosis and assessment of disease progression. Orphanet J Rare Dis. 2021;16:411. doi: 10.1186/s13023-021-01960-9 34602081 PMC8489116

[17] Latini R , Masson S , Anand IS , Missov E , Carlson M , Vago T , Angelici L , Barlera S , Parrinello G , Maggioni AP , et al. Prognostic value of very low plasma concentrations of troponin T in patients with stable chronic heart failure. Circulation. 2007;116:1242–1249. doi: 10.1161/CIRCULATIONAHA.106.655076 17698733

[18] Berry C , Poppe KK , Gamble GD , Earle NJ , Ezekowitz JA , Squire IB , McMurray JJV , McAlister FA , Komajda M , Swedberg K , et al. Prognostic significance of anaemia in patients with heart failure with preserved and reduced ejection fraction: results from the MAGGIC individual patient data meta‐analysis. QJM. 2016;109:377–382. doi: 10.1093/qjmed/hcv087 25979270 PMC5943826

[19] Kalra PR , Cleland JGF , Petrie MC , Thomson EA , Kalra PA , Squire IB , Ahmed FZ , Al‐Mohammad A , Cowburn PJ , Foley PWX , et al. Intravenous ferric derisomaltose in patients with heart failure and iron deficiency in the UK (IRONMAN): an investigator‐initiated, prospective, randomised, open‐label, blinded‐endpoint trial. Lancet. 2022;400:2199–2209. doi: 10.1016/S0140-6736(22)02083-9 36347265

[20] Anker SD , Comin Colet J , Filippatos G , Willenheimer R , Dickstein K , Drexler H , Lüscher TF , Bart B , Banasiak W , Niegowska J , et al. Ferric carboxymaltose in patients with heart failure and iron deficiency. N Engl J Med. 2009;361:2436–2448. doi: 10.1056/NEJMoa0908355 19920054

[21] Maurer MS , Schwartz JH , Gundapaneni B , Elliott PM , Merlini G , Waddington‐Cruz M , Kristen AV , Grogan M , Witteles R , Damy T , et al. Tafamidis treatment for patients with transthyretin amyloid cardiomyopathy. N Engl J Med. 2018;379:1007–1016. doi: 10.1056/NEJMoa1805689 30145929

[22] Kazi DS , Bellows BK , Baron SJ , Shen C , Cohen DJ , Spertus JA , Yeh RW , Arnold SV , Sperry BW , Maurer MS , et al. Cost‐effectiveness of tafamidis therapy for transthyretin amyloid cardiomyopathy. Circulation. 2020;141:1214–1224. doi: 10.1161/CIRCULATIONAHA.119.045093 32078382 PMC7156331

[23] Ioannou A , Massa P , Patel RK , Razvi Y , Porcari A , Rauf MU , Jiang A , Cabras G , Filisetti S , Bolhuis RE , et al. Conventional heart failure therapy in cardiac ATTR amyloidosis. Eur Heart J. 2023;44:2893–2907. doi: 10.1093/eurheartj/ehad347 37216684 PMC10424879

[24] Garcia‐Pavia P , Rapezzi C , Adler Y , Arad M , Basso C , Brucato A , Burazor I , Caforio ALP , Damy T , Eriksson U , et al. Diagnosis and treatment of cardiac amyloidosis: a position statement of the ESC Working Group on Myocardial and Pericardial Diseases. Eur Heart J. 2021;42:1554–1568. doi: 10.1093/eurheartj/ehab072 33825853 PMC8060056

[25] Writing Committee , Kittleson MM , Ruberg FL , Ambardekar AV , Brannagan TH , Cheng RK , Clarke JO , Dember LM , Frantz JG , Hershberger RE , et al. ACC expert consensus decision pathway on comprehensive multidisciplinary care for the patient with cardiac amyloidosis: a report of the American College of Cardiology Solution Set Oversight Committee. J Am Coll Cardiol. 2023;81:1076–1126. doi: 10.1016/j.jacc.2022.11.022 36697326

