# Supplementary material for: Multiorgan Dysfunction and Associated Prognosis in Transthyretin Cardiac Amyloidosis
Source: J Am Heart Assoc. 2024 Feb 5;13(4):e033094. doi: 10.1161/JAHA.123.033094 (PMC11010096; doi:10.1161/JAHA.123.033094)
Supplement: Supplementary file 1 — Tables S1–S11. [file JAH3-13-e033094-s001.pdf]

Supplemental Material

Table S1. Normal laboratory ranges for full blood count and serum biochemistry variables.

Blood biomarkers	Normal range
**Full blood count**	
Haemoglobin (g/L)	Male: 135 – 170<br>Female: 110 – 150
WBC x 10^9/L	3.5 – 11.0
Platelets x 10^9/L	140 - 400
**Serum biochemistry**	
Sodium (mmol/L)	135 - 145
Potassium (mmol/L)	3.5 – 5.1
Urea (mmol/L)	2.9 – 8.1
Creatinine (µmol/L)	66 – 112
eGFR (ml/min/1.73m^2)	> 90
Total bilirubin (µmol/L)	> 21
Alanine transaminase (IU/L)	Male: 10 – 50<br>Female: 10 – 35
Aspartate aminotransferase (IU/L)	Male: 10 – 50<br>Female: 10 – 35
Alkaline phosphatase (IU/L)	> 129
Gamma GT (IU/L)	5 – 36
Total protein (g/L)	66 – 87
Albumin (g/L)	35 – 50
C-reactive protein (mg/L)	< 5

Table S2. Baseline characteristics and blood biomarkers for the whole ATTR-CA population, and split by genotype into patients with wtATTR-CA, p.(V142I)-hATTR-CA and non-p.(V142I)-hATTR-CA.

	wtATTR-CA (n = 1834, 71.5%)	p.(V142I)-hATTR-CA (n = 425, 16.6%)	Non-p.(V142I)-hATTR-CA (n = 307, 12.0%)	wt vs p.(V142I) standardised differences	wt vs non-p.(V142I) standardised differences	p.(V142I) vs non-p.(V142I) standardised differences
**Demographics**						
Age (years)	78.8 ± 6.6	76.4 ± 7.3	66.4 ± 9.8	0.348	1.489	1.158
Sex (male)	1715 (93.5%)	288 (67.8%)	215 (70.0%)	0.689	0.6375	-0.049
**Cardiac biomarkers**						
NAC stage						
1	866 (47.2%)	191 (44.9%)	210 (68.4%)	0.045	-0.439	-0.487
2	664 (36.2%)	146 (34.4%)	78 (25.4%)	0.039	0.235	0.196
3	304 (16.6%)	88 (20.7%)	19 (6.2%)	-0.106	0.331	0.435
NT-proBNP (ng/L)	2907 (1499 – 5281)	2850 (1548 – 5704)	1852 (626 – 3714)	-0.087	0.248	0.314
Troponin-T (ng/L)	58 (40 – 84)	73 (49 – 108)	40 (25 – 60)	-0.319	0.418	0.668
Troponin-T >14ng/L	1742 (98.9%)	395 (99.0%)	260 (92.5%)	-0.013	0.316	0.325
Troponin-T>56ng/L	915 (51.9%)	266 (66.7%)	82 (29.2%)	-0.305	0.475	0.808
**Full blood count**						
Haemoglobin (g/L)	137 (126 – 148)	129 (120 – 140)	134 (124 – 144)	0.447	0.187	-0.295
Anaemia	716 (39.0%)	179 (42.1%)	95 (30.9%)	-0.063	0.170	0.233
WBC x 10^9/L	6.8 (5.8 – 6.9)	5.0 (4.2 – 6.1)	6.3 (5.2 – 7.5)	0.855	0.306	-0.610
Platelets x 10^9/L	202 (170 – 240)	190 (156 – 233)	208 (179 – 253)	0.158	-0.182	-0.328
Low platelets	263 (14.4%)	91 (21.4%)	33 (10.8%)	-0.184	0.109	0.292
**Serum biochemistry**						
Sodium (mmol/L)	141 (139 – 143)	141 (139 – 143)	141 (139 – 143)	-0.168	0.013	0.179
Hyponatraemia	114 (6.2%)	15 (3.5%)	22 (7.2%)	0.125	-0.038	-0.162
Potassium (mmol/L)	4.5 (4.3 – 4.8)	4.5 (4.2 – 4.8)	4.4 (4.2 – 4.7)	0.173	0.326	0.134
Urea (mmol/L)	8.9 (6.9 – 11.7)	8.0 (6.2 – 11.0)	6.4 (5.2 – 8.3)	0.108	0.667	0.501
High-urea	1061 (57.9%)	201 (47.3%)	80 (26.1%)	0.212	0.680	0.451
Creatinine (µmol/L)	107 (90 – 131)	110 (92 – 133)	81 (68 – 98)	-0.079	0.571	0.617
eGFR (ml/min/1.73m²)	59 (46 – 72)	55 (43 – 67)	80 (64 – 90)	0.256	-0.908	-1.181
eGFR < 60 ml/min/1.73m²	953 (52.0%)	261 (61.4%)	57 (18.6%)	-0.192	0.745	0.971
Serum total bilirubin (µmol/L)	12 (10 – 19)	13 (8 – 20)	11 (8 – 15)	-0.070	0.355	0.377
Hyperbilirubinemia	337 (18.4%)	89 (20.9%)	33 (10.7%)	-0.065	0.217	0.281
Alanine transaminase (IU/L)	24 (19 – 31)	27 (20 – 37)	24 (19 – 32)	-0.246	-0.072	0.164
High alanine transaminase	99 (5.4%)	60 (14.1%)	33 (10.7%)	-0.297	-0.197	0.102
Aspartate aminotransferase (IU/L)	29 (24 – 35)	32 (26 – 42)	26 (21 – 31)	-0.265	0.215	0.382
High aspartate aminotransferase	84 (4.6%)	75 (17.6%)	19 (6.2%)	-0.297	-0.071	0.359
Alkaline phosphatase (IU/L)	94 (74 – 143)	97 (76 – 134)	78 (62 – 103)	-0.069	0.375	0.444
High alkaline phosphatase	323 (17.6%)	80 (18.8%)	18 (5.9%)	-0.031	0.371	0.401
GGT (IU/L)	69 (34 – 143)	104 (51 – 209)	33 (18 – 75)	-0.294	0.440	0.716
High GGT	1308 (71.3%)	350 (82.4%)	133 (43.3%)	-0.264	0.590	0.882
Total protein (g/L)	71 (67 – 74)	73 (69 – 76)	68 (65 – 72)	-0.329	0.503	0.799
Albumin (g/L)	44 (42 – 46)	42 (40 – 45)	43 (41 – 46)	0.449	0.247	-0.204
Hypoalbuminemia	16 (0.9%)	17 (4.0%)	5 (1.6%)	-0.204	-0.068	0.144

C-reactive protein (mg/L)	2 (1 – 5)	3 (1 – 7)	1 (1 – 3)	-0.150	0.089	0.205
High c-reactive protein	509 (27.8%)	139 (32.7%)	52 (16.9%)	-0.108	0.262	0.371
**Echocardiographic parameters**						
IVSd (mm)	16.9 ± 2.5	16.8 ± 2.3$^{\gamma}$	16.2 ± 3.1	0.058	0.242	0.197
PWTd (mm)	16.4 ± 2.6	16.5 ± 2.4	15.9 ± 3.1	-0.014	0.189	0.209
RWT	0.77 ± 0.17	0.81 ± 0.18	0.79 ± 0.19	-0.184	-0.094	0.083
LVEF (%)	48.9 ± 10.2	43.7 ± 11.0	50.8 ± 10.9	0.483	-0.189	-0.648
LVEF ≤40%	377 (20.6%)	169 (39.8%)	52 (16.9%)	-0.428	0.093	0.522
Stroke volume (ml)	38.7 ± 13.4	30.8 ± 11.8	36.8 ± 12.7	0.626	0.143	-0.490
Longitudinal strain (%)	-11.1 ± 3.7	-9.7 ± 3.3	-11.9 ± 4.3	-0.400	0.204	0.581
Average E/e’	16.4 ± 6.3	17.9 ± 6.4	17.4 ± 7.7	-0.233	-0.343	0.071

P-values for pairwise comparison: α = P<0.05 for wtATTR vs. p.(V142I) hATTR, β = P<0.05 for wtATTR vs. non-p.(V142I) hATTR, γ = P<0.05 for p.(V142I) hATTR vs. non-p.(V142I) hATTR

wtATTR-CA = Wild-type transthyretin cardiac amyloidosis; hATTR-CA = Hereditary transthyretin cardiac amyloidosis; WBC = White blood count; NAC = National Amyloidosis Centre; NT-pro-BNP = N-terminal pro B-type natriuretic peptide; eGFR = Estimated glomerular filtration rate; GGT = gamma-glutamyl transferase; IVSd = Interventricular septal thickness in diastole; PWTd = Posterior wall thickness in diastole; RWT = Relative wall thickness; LVEF = Left ventricular ejection fraction.

Table S3. Blood biomarkers split by National Amyloidosis Centre disease stage.

	NAC stage 1 (n = 1267, 49.4%)	NAC stage 2 (n = 888, 34.6%)	NAC stage 3 (n = 411, 16.0%)	1 vs 2 standardised differences	1 vs 3 standardised differences	2 vs 3 standardised differences
**Full blood count**						
Haemoglobin (g/L)	138 (128 – 148)	135 (124 – 146)	126 (115 – 139)	0.204	0.632	0.408
Anaemia	390 (30.8%)	367 (41.3%)	233 (56.7%)	-0.221	-0.541	-0.311
WBC x 10^9/L	6.5 (5.4 – 7.8)	6.6 (5.4 – 7.9)	6.4 (5.2 – 7.8)	-0.059	0.008	0.063
Platelets x 10^9/L	206 (175 – 247)	197 (162 – 238)	189 (156 – 232)	0.146	0.283	0.125
Low platelets	137 (10.8%)	162 (18.3%)	88 (21.5%)	-0.214	-0.292	-0.078
**Serum biochemistry**						
Sodium (mmol/L)	141 (139 – 143)	141 (138 – 143)	140 (138 – 142)	0.122	0.230	0.098
Hyponatraemia	54 (4.3%)	67 (7.5%)	30 (7.3%)	-0.140	-0.130	0.009
Potassium (mmol/L)	4.5 (4.3 – 4.8)	4.5 (4.2 – 4.8)	4.6 (4.3 – 5.0)	-0.016	-0.238	-0.208
Urea (mmol/L)	7.1 (5.8 – 8.7)	9.3 (7.3 – 11.6)	14.5 (11.5 – 19.2)	-0.754	-1.770	-1.200
High-urea	397 (31.3%)	557 (62.7%)	388 (94.4%)	-0.662	-1.722	-0.836
Creatinine (μmol/L)	93 (79 – 107)	110 (94 – 126)	160 (144 – 184)	-0.591	-2.069	-1.289
Serum total bilirubin (μmol/L)	11 (8 – 11)	15 (10 – 21)	14 (10 – 21)	-0.370	-0.329	0.032
Hyperbilirubinemia	155 (12.2%)	209 (23.5%)	95 (32.1%)	-0.298	-0.288	0.010
Alanine transaminase (IU/L)	25 (19 – 33)	25 (20 – 34)	23 (18 – 30)	-0.034	0.083	0.120
High alanine transaminase	90 (7.1%)	71 (8.0%)	31 (7.5%)	-0.034	-0.017	0.017
Aspartate aminotransferase (IU/L)	28 (23 – 34)	30 (25 – 37)	29 (24 – 37)	-0.255	-0.190	-0.043
High aspartate aminotransferase	67 (5.3%)	66 (7.4%)	45 (10.9%)	-0.034	-0.208	-0.122
Alkaline phosphatase (IU/L)	83 (67 – 106)	102 (77 – 137)	114 (87 – 155)	-0.419	-0.609	-0.252
High alkaline phosphatase	115 (9.1%)	190 (21.4%)	116 (28.2%)	-0.348	-0.507	-0.158
GGT (IU/L)	48 (26 – 107)	88 (44 – 184)	108 (55 – 210)	-0.358	-0.501	-0.191
High GGT	738 (58.2%)	707 (79.6%)	346 (84.2%)	-0.474	-0.598	-0.119
Albumin (g/L)	44 (42 – 46)	43 (41 – 45)	43 (41 – 45)	0270	0.350	0.081
Hypoalbuminemia	9 (0.7%)	17 (1.9%)	12 (2.9%)	-0.106	-0.166	-0.065
C-reactive protein (mg/L)	2 (1 – 4)	3 (1 – 6)	4 (2 – 8)	-0.195	-0.331	-0.133
High c-reactive protein	242 (19.1%)	281 (31.6%)	177 (43.1%)	-0.291	-0.536	-0.238
NT-proBNP (ng/L)	1468 (846 – 2155)	4533 (3374 – 6494)	6952 (4703 – 10981)	-1.306	-1.548	-0.669
Troponin-T (ng/L)	43 (29 – 60)	69 (51 – 94)	104 (74 – 146)	-0.680	-1.146	-0.603
Troponin-T > 14ng/L	1163 (96.5%)	841 (99.6%)	393 (100.0%)	-0.229	-0.269	-0.084
Troponin-T > 56ng/L	355 (29.5%)	565 (66.9%)	342 (87.0%)	-0.809	-1.436	-0.491
**Echocardiographic parameters**						
IVSd (mm)	16.3 ± 2.6	17.3 ± 2.4	17.2 ± 2.4	-0.391	-0.324	0.065
PWTd (mm)	15.8 ± 2.6	16.9 ± 2.4	16.8 ± 2.6	-0.432	-0.354	0.066
RWT	0.75 ± 0.17	0.80 ± 0.18	0.81 ± 0.19	-0.334	-0.403	-0.077
LVEF (%)	51.0 ± 9.5	45.8 ± 10.7	44.9 ± 11.3	0.507	0.578	0.081
LVEF ≤40%	185 (14.6%)	270 (30.4%)	143 (34.8%)	-0.385	-0.481	-0.094
Stroke volume (ml)	39.7 ± 13.7	35.5 ± 12.5	33.0 ± 12.9	0.322	0.509	0.202
Longitudinal strain (%)	-12.2 ± 3.8	-9.8 ± 3.2	-9.5 ± 3.3	-0.696	-0.784	-0.099
Average E/e’	15.6 ± 5.9	17.9 ± 6.9	18.1 ± 6.9	-0.363	-0.400	-0.035

P-values for pairwise comparison: α = P<0.01 for NAC stage 1 vs. NAC stage 2, β = P<0.01 NAC stage 1 vs. NAC stage 3, γ = P<0.01 NAC stage 2 vs NAC stage 3.

WBC = White blood count; NAC = National Amyloidosis Centre; NT-pro-BNP = N-terminal pro B-type natriuretic peptide; eGFR = Estimated glomerular filtration rate; GGT = gamma-glutamyl transferase; IVSd = Interventricular septal thickness in diastole; PWTd = Posterior wall thickness in diastole; RWT = Relative wall thickness; LVEF = Left ventricular ejection fraction.

Table S4. Blood biomarkers split by NYHA class.

	NYHA class I (n = 1267,	NYHA class II (n = 1531,	NYHA class III (n = 515	NYHA class IV (n = 38	P-value
**Full blood count**					
Haemoglobin (g/L)	137 (126 – 147)$^{\beta\gamma}$	137 (126 – 147)$^{\delta\varepsilon}$	132 (120 – 143)$^{\phi}$	127 (111 – 134)	<0.001
Anaemia	111 (34.2%)$^{\beta\gamma}$	570 (37.2%)$^{\delta\varepsilon}$	226 (43.9%)	23 (60.5%)	<0.001
WBC x 10^9/L	6.5 (5.3 – 7.7)$^{\gamma}$	6.6 (5.4 – 7.9)$^{\delta\varepsilon}$	6.2 (5.1 – 7.6)$^{\phi}$	5.2 (4.1 – 6.9)	<0.001
Platelets x 10^9/L	204 (174 – 254)$^{\delta}$	201 (167 – 239) $^{\varepsilon}$	197 (166 – 231)$^{\phi}$	191 (161 – 244)	0.022
Low platelets	37 (11.4%)	238 (15.6%)	87 (17.0%)	4 (10.5%)	0.126
**Serum biochemistry**					
Sodium (mmol/L)	141 (139 – 143)	141 (139 – 143)	141 (138 – 143)	141 (139 – 143)	0.339
Hyponatraemia	15 (4.6%)	78 (5.1%)	38 (7.4%)	2 (5.3%)	0.216
Potassium (mmol/L)	4.5 (4.2 – 4.8)	4.5 (4.2 – 4.8)	4.5 (4.2 – 4.9)	4.3 (4.0 – 4.6)	0.007
Urea (mmol/L)	6.9 (5.6 – 8.6)$^{\alpha\beta\gamma}$	8.5 (6.6 – 11.1)$^{\delta\varepsilon}$	9.6 (7.2 – 12.8)	10.9 (7.2 – 16.3)	<0.001
Uraemia	94 (28.9%)$^{\alpha\beta\gamma}$	800 (52.3%)$^{\delta}$	331 (64.3%)	26 (68.4%)	<0.001
Creatinine (μmol/L)	93 (79 – 111)$^{\alpha\beta\gamma}$	105 (87 – 127)$^{\delta\varepsilon}$	113 (94 – 141)	140 (103 – 176)	<0.001
Serum total bilirubin (μmol/L)	11 (8 – 16)$^{\alpha\beta\gamma}$	13 (9 – 18)$^{\delta}$	14 (9 – 21)	18 (11 – 23)	<0.001
Hyperbilirubinemia	37 (11.4%)$^{\beta\gamma}$	263 (17.2%)$^{\delta}$	122 (23.7%)	27 (71.1%)	<0.001
Alanine transaminase (IU/L)	25 (19 – 33)$^{\beta\gamma}$	25 (19 – 33)	24 (18 – 32)	19 (13 – 26)	<0.001
High alanine transaminase	22 (6.8%)	108 (7.1%)	51 (9.9%)	1 (2.6%)	0.101
Aspartate aminotransferase (IU/L)	27 (22 – 34)$^{\gamma}$	29 (24 – 36)$^{\varepsilon}$	29 (24 – 36)$^{\phi}$	31 (24 – 39)	0.004
High aspartate aminotransferase	17 (5.2%)	94 (6.1%)$^{\delta}$	53 (10.3%)	4 (10.5%)	0.005
Alkaline phosphatase (IU/L)	81 (64 – 105)$^{\alpha\beta\gamma}$	92 (73 – 139)$^{\delta\varepsilon}$	102 (77 – 142)	119 (89 – 164)	<0.001
High alkaline phosphatase	24 (7.4%)$^{\alpha\beta\gamma}$	243 (15.9%)$^{\delta}$	114 (77.9%)	12 (31.6%)	<0.001
GGT (IU/L)	40 (23 – 102)$^{\alpha\beta\gamma}$	69 (33 – 139)$^{\delta\varepsilon}$	97 (44 – 184)	104 (53 – 212)	<0.001
High GGT	171 (52.6%)$^{\alpha\beta\gamma}$	1085 (70.9%)$^{\delta}$	402 (78.1%)	32 (84.2%)	<0.001
Albumin (g/L)	44 (42 – 46)$^{\beta\gamma}$	44 (42 – 46)$^{\delta\varepsilon}$	43 (41 – 45)$^{\phi}$	41 (38 – 43)	<0.001
Hypoalbuminemia	1 (0.3%)$^{\beta\gamma}$	13 (0.8%)$^{\delta\varepsilon}$	15 (2.9%)	3 (7.9%)	<0.001
C-reactive protein (mg/L)	2 (1 – 3)$^{\alpha\beta\gamma}$	2 (1 – 5)$^{\delta\varepsilon}$	3 (1 – 6)$^{\phi}$	7 (4 – 13)	<0.001
High c-reactive protein	59 (18.2%)$^{\alpha\beta\gamma}$	386 (25.2%)$^{\delta\varepsilon}$	181 (35.1%)$^{\phi}$	26 (68.4%)	<0.001
NT-proBNP (ng/L)	1438 (578 – 2715) $^{\alpha\beta\gamma}$	2605 (1404 – 4716) $^{\delta\varepsilon}$	4203 (2162 – 7476)	5918 (3445 – 10351)	<0.001
Troponin-T (ng/L)	42 (26 – 64)$^{\alpha\beta\gamma}$	57 (38 – 81)$^{\delta\varepsilon}$	75 (49 – 109)	106 (69 – 168)	<0.001
Troponin-T > 14ng/L	290 (93.5%)$^{\alpha\beta}$	1450 (98.6%)	484 (99.4%)	29 (96.7%)	<0.001
Troponin-T > 56ng/L	95 (30.6%)$^{\alpha\beta\gamma}$	743 (50.5%)$^{\delta\varepsilon}$	318 (65.3%)	24 (80.0%)	<0.001
**Echocardiographic parameters**					
IVSd (mm)	16.3 ± 2.6$^{\alpha\beta}$	16.9 ± 2.5	17.1 ± 2.2	17.4 ± 2.3	<0.001
PWTd (mm)	15.8 ± 2.6$^{\alpha\beta\gamma}$	16.5 ± 2.5	16.7 ± 2.4	17.1 ± 2.2	<0.001
LVEF (%)	51.0 ± 9.5$^{\alpha\beta\gamma}$	48.7 ± 10.2$^{\delta\varepsilon}$	44.1 ± 11.2	40.5 ± 10.5	<0.001
LVEF ≤40%	33 (10.2%)$^{\alpha\beta}$	326 (21.3%)$^{\delta\varepsilon}$	195 (37.9)	18 (47.4%)	<0.001
Longitudinal strain (%)	-12.3 ± 3.8$^{\alpha\beta\gamma}$	-11.1 ± 3.7$^{\delta\varepsilon}$	-9.8 ± 3.4$^{\phi}$	-7.9 ± 2.9	<0.001
Average E/e’	15.6 ± 5.9$^{\alpha\beta}$	16.6 ± 6.4$^{\delta}$	18.1 ± 7.0	18.4 ± 8.6	<0.001

P-values for pairwise comparison: α = P<0.05 for NYHA class I vs. NYHA class II, β = P<0.05 for NYHA class I vs. NYHA class III, γ = P<0.05 for NYHA class I vs. NYHA class IV, δ = 0.05 for NYHA class II vs. NYHA class III, ε = NYHA class II vs. NYHA class IV, ϕ = NYHA class III vs. NYHA class IV

NYHA = New York Heart Association; WBC = White blood count; NAC = National Amyloidosis Centre; NT-pro-BNP = N-terminal pro B-type natriuretic peptide; eGFR = Estimated glomerular filtration rate; GGT = gamma-glutamyl transferase; IVSd = Interventricular septal thickness in diastole; PWTd = Posterior wall thickness in diastole; LVEF = Left ventricular ejection fraction.

Table S5. Blood biomarkers split by left ventricular ejection fraction.

	LVEF ≤40% (n=598, 23.3%)	LVEF >40% (n=1968, 76.7%)	P-value
**Full blood count**			
Haemoglobin (g/L)	136 (123 – 146)	135 (125 – 146)	0.283
Anaemia	229 (38.3%)	761 (38.7%)	0.869
WBC x 10^9/L	6.4 (5.1 – 7.8)	6.6 (5.4 – 7.8)	0.014
Platelets x 10^9/L	196 (161 – 241)	141 (139 – 143)	0.050
Low platelets	106 (17.3%)	281 (14.3%)	0.041
**Serum biochemistry**			
Sodium (mmol/L)	141 (138 – 143)	141 (139 – 143)	0.525
Hyponatraemia	40 (6.7%)	111 (5.6%)	0.340
Potassium (mmol/L)	4.5 (4.2 – 4.8)	4.5 (4.2 – 4.8)	0.915
Urea (mmol/L)	9.2 (7.0 – 11.9)	8.2 (6.4- 10.9)	<0.001
Uraemia	365 (61.0%)	977 (49.6%)	<0.001
Creatinine (μmol/L)	112 (93 – 137)	103 (86 – 125)	<0.001
eGFR (ml/min/1.73m²)	55 (44 – 69)	61 (48 – 76)	<0.001
eGFR < 60 ml/min/1.73m²	352 (58.9%)	919 (46.7%)	<0.001
Serum total bilirubin (μmol/L)	15 (11 – 22)	12 (9 – 18)	<0.001
Hyperbilirubinemia	158 (26.4%)	301 (15.3%)	<0.001
Alanine transaminase (IU/L)	25 (19 – 34)	24 (9 – 18)	0.604
High alanine transaminase	56 (9.4%)	136 (6.9%)	0.046
Aspartate aminotransferase (IU/L)	30 (25 – 38)	28 (19 – 32)	<0.001
High aspartate aminotransferase	66 (11.0%)	112 (5.7%)	<0.001
Alkaline phosphatase (IU/L)	101 (78 – 143)	90 (71 – 120)	<0.001
High alkaline phosphatase	140 (23.4%)	281 (14.3%)	<0.001
GGT (IU/L)	96 (47 – 207)	62 (30 – 127)	<0.001
High GGT	481 (80.4%)	1310 (66.6%)	<0.001
Albumin (g/L)	43 (41 – 45)	44 (42 – 46)	<0.001
Hypoalbuminemia	12 (2.0%)	26 (1.3%)	0.224
C-reactive protein (mg/L)	3 (1 – 7)	2 (1 – 3)	<0.001
High c-reactive protein	202 (33.8%)	498 (25.3%)	<0.001
NT-proBNP (ng/L)	4114 (2466 – 7201)	2379 (1219 – 4521)	<0.001
Troponin-T (ng/L)	73 (48 – 104)	54 (37 – 79)	<0.001
Troponin-T > 14ng/L	572 (99.7%)	1827 (97.7%)	0.002
Troponin-T > 56ng/L	375 (65.6%)	887 (47.4%)	<0.001
**Echocardiographic parameters**			
IVSd (mm)	17.0 ± 2.5	16.7 ± 2.6	0.026
PWTd (mm)	16.6 ± 2.6	16.3 ± 2.6	0.016
LVEF (%)	33.6 ± 5.4	52.9 ± 7.1	<0.001
Longitudinal strain (%)	-8.0 ± 2.6	-11.9 ± 3.6	<0.001
Average E/e’	18.1 ± 7.1	16.4 ± 6.3	<0.001

LVEF = Left ventricular ejection fraction;WBC = White blood count; NT-pro-BNP = N-terminal pro B-type natriuretic peptide; eGFR = Estimated glomerular filtration rate; GGT = gamma-glutamyl transferase; IVSd = Interventricular septal thickness in diastole; PWTd = Posterior wall thickness in diastole; LVEF = Left ventricular ejection fraction

Table S6. Correlation between stroke volume and blood biomarkers.

Correlation with stroke volume (ml)		
	Correlation coefficient	P-value
Haemoglobin (g/L)	0.011	0.620
Sodium (mmol/L)	0.016	0.481
Urea (mmol/L)	-0.102	<0.001
Serum total bilirubin (μmol/L)	-0.158	<0.001
Alanine transaminase (IU/L)	-0.005	<0.810
Aspartate aminotransferase (IU/L)	-0.178	<0.001
Alkaline phosphatase (IU/L)	-0.162	<0.001
GGT (IU/L)	-0.181	<0.001
Albumin (g/L)	0.132	<0.001
C-reactive protein (mg/L)	-0.132	<0.001
Troponin-T (ng/L)	-0.174	<0.001

Table S7. Multivariable linear regression assessing the independent relationship between blood biomarkers and stroke volume.

Multivariable linear regression model		
**Stroke volume (ml)**	**Coefficient 95%(CI)**	**P-value**
NAC stage 1 disease	Ref	
NAC stage 2 disease	-3.38 (-4.79 to -1.97)	<0.001
NAC stage 3 disease	-6.14 (-8.38 to -3.90)	<0.001
Haemoglobin (g/L)	-0.03 (-0.07 to 0.01)	0.091
Sodium (mmol/L)	-0.11 (-0.29 to 0.06)	0.210
Urea (mmol/L)	0.12 (-0.05 to 0.29)	0.168
Serum total bilirubin (μmol/L)	-0.14 (-0.21 to -0.07)	<0.001
Alanine transaminase (IU/L)	0.00 (-0.04 to 0.04)	0.998
Alkaline phosphatase (IU/L)	0.00 (-0.02 to 0.01)	0.834
GGT (IU/L)	-0.01 (-0.01 to 0.00)	0.070
Albumin (g/L)	0.43 (0.24 to 0.62)	<0.001
C-reactive protein (mg/L)	-0.02 (-0.08 to 0.03)	0.396
Troponin-T (ng/L)	-0.01 (-0.02 to 0.01)	0.227

NAC = National Amyloidosis Centre; GGT = gamma-glutamyl transferase.

Table S8. Univariable Cox proportional hazards regression analysis of risk of death in the overall ATTR-CA population.

Univariable Cox regression of continuous variables		
	**HR (95%CI)**	**P-value**
Haemoglobin (g/L)	0.99 (0.98-0.99)	<0.001
Platelets x 10^9/L	1.00 (0.99-1.00)	0.006
Sodium (mmol/L)	0.94 (0.93-0.96)	<0.001
Urea (mmol/L)	1.10 (1.09-1.11)	<0.001
Total bilirubin (μmol/L)	1.02 (1.02-1.03)	<0.001
ALT (IU/L)	1.00 (0.99-1.00)	0.307
AST (IU/L)	1.01 (1.01-1.01)	<0.001
ALP (IU/L)	1.00 (1.00-1.01)	<0.001
GGT (IU/L)	1.00 (1.00-1.00)	<0.001
Albumin (g/L)	0.93 (0.91-0.94)	<0.001
CRP (mg/L)	1.01 (1.01-1.01)	<0.001
Troponin-T (ng/L)	1.01 (1.00-1.01)	<0.001
**Univariable Cox regression of z-score standardised continuous variables**		
	**HR (95%CI)**	**P-value**
Haemoglobin (g/L)	0.79 (0.74-0.84)	<0.001
Platelets x 10^9/L	0.91 (0.85-0.97)	0.006
Sodium (mmol/L)	0.82 (0.77-0.87)	<0.001
Urea (mmol/L)	1.56 (1.49-1.64)	<0.001
Total bilirubin (μmol/L)	1.22 (1.16-1.28)	<0.001
ALT (IU/L)	0.97 (0.91-1.03)	0.307
AST (IU/L)	1.14 (1.09-1.18)	<0.001
ALP (IU/L)	1.28 (1.22-1.34)	<0.001
GGT (IU/L)	1.26 (1.20-1.32)	<0.001
Albumin (g/L)	0.77 (0.72-0.82)	<0.001
CRP (mg/L)	1.13 (1.08-1.18)	<0.001
Troponin-T (ng/L)	1.32 (1.29-1.36)	<0.001
**Univariable Cox regression of dichotomous variables**		
	**HR (95%CI)**	**P-value**
Anaemia	1.59 (1.40-1.80)	<0.001
Low platelets	1.25 (1.05-1.48)	0.010
Hyponatraemia	1.80 (1.42-2.28)	<0.001
Uraemia	2.06 (1.8-2.36)	<0.001
Hyperbilirubinemia	1.77 (1.5-2.05)	<0.001
High ALT	0.99 (0.78-1.25)	0.915
High AST	1.55 (1.24-1.95)	<0.001
High ALP	1.98 (1.71-2.30)	<0.001
High GGT	1.70 (1.46-1.98)	<0.001
Hypoalbuminemia	3.39 (2.31-4.97)	<0.001
High CRP	1.66 (1.45-1.89)	<0.001
Troponin-T >56ng/L	2.75 (2.36-3.19)	<0.001

HR = Hazard ratio; CI = Confidence interval; ALT = alanine transaminase; AST = Aspartate aminotransferase; ALP = Alkaline phosphatase; GGT = gamma-glutamyl transferase; CRP = C-reactive protein.

Table S9. Multivariable Cox proportional hazards regression analysis of risk of death in the overall ATTR-CA population.

Multivariable Cox regression analysis of z-score standardised continuous variables					
Patients not censored for the start date of clinical trials and disease modifying therapy			Patients censored for the start date of clinical trials and disease modifying therapy		
**Variable**	**HR (95%CI)**	**P-value**	**Variable**	**HR (95%CI)**	**P-value**
Haemoglobin (g/L)	0.88 (0.2-0.94)	<0.001	Haemoglobin (g/L)	0.88 (0.81-0.94)	0.001
Sodium (mmol/L)	0.90 (0.84-0.96)	0.003	Sodium (mmol/L)	0.92 (0.86-0.99)	0.021
Urea (mmol/L)	1.32 (1.24-1.41)	<0.001	Urea (mmol/L)	1.30 (1.22-1.39)	<0.001
Total bilirubin (μmol/L)	1.15 (1.09-1.22)	<0.001	Total bilirubin (μmol/L)	1.15 (1.08-1.22)	<0.001
ALP (IU/L)	1.00 (0.92-1.08)	0.933	ALP (IU/L)	1.00 (0.92-1.08)	0.906
GGT (IU/L)	1.06 (0.97-1.14)	0.183	GGT (IU/L)	1.05 (0.96-1.14)	0.287
Albumin (g/L)	0.88 (0.820.94)	<0.001	Albumin (g/L)	0.88 (0.82-0.95)	0.001
CRP (mg/L)	1.03 (0.97-1.10)	0.274	CRP (mg/L)	1.04 (0.98-1.10)	0.250
Troponin-T (ng/L)	1.24 (1.19-1.29)	<0.001	Troponin-T (ng/L)	1.24 (1.19-1.29)	<0.001
Harrell's c	0.712 (0.694-0.731)	<0.001	Harrell's c	0.711 (0.691-0.731)	<0.001

HR = Hazard ratio; CI = Confidence interval; ALP = Alkaline phosphatase; GGT = gamma-glutamyl transferase; CRP = C-reactive protein.

Table S10. Multivariable Cox proportional hazards regression analyses of risk of death in the overall ATTR-CA population adjusting for furosemide equivalent dose and stroke volume.

**Multivariable Cox regression analysis in the overall population adjusting for furosemide dose**					
**Patients not censored for the start date of clinical trials and disease modifying therapy**			**Patients censored for the start date of clinical trials and disease modifying therapy**		
**Variable**	**HR (95%CI)**	**P-value**	**Variable**	**HR (95%CI)**	**P-value**
Furosemide (mg/24hrs)	1.00 (1.00-1.00)	<0.001	Furosemide (mg/24hrs)	1.00 (1.00-1.00)	<0.001
Anaemia	1.22 (1.06-1.40)	0.004	Anaemia	1.20 (1.04-1.38)	0.015
Hyponatraemia	1.66 (1.29-2.13)	<0.001	Hyponatraemia	1.72 (1.32-2.22)	<0.001
Uraemia	1.36 (1.16-1.58)	<0.001	Uraemia	1.36 (1.15-1.59)	<0.001
Hyperbilirubinemia	1.30 (1.10-1.53)	0.002	Hyperbilirubinemia	1.28 (1.08-1.51)	0.005
High ALP	1.20 (1.01-1.42)	0.041	High ALP	1.18 (0.98-1.41)	0.067
High GGT	1.13 (0.95-1.33)	0.164	High GGT	1.11 (0.93-1.33)	0.237
Hypoalbuminemia	1.52 (0.99-2.33)	0.054	Hypoalbuminemia	1.57 (1.02-2.40)	0.039
High CRP	1.17 (1.02-1.36)	0.030	High CRP	1.15 (0.99-1.34)	0.064
Troponin-T >56ng/L	1.99 (1.68-2.33)	<0.001	Troponin-T >56ng/L	1.90 (1.60-2.25)	<0.001
Harrell's c	0.700 (0.682-0.718)	<0.001	Harrell's c	0.698 (0.79-0.717)	<0.001
**Multivariable Cox regression analysis in the overall population adjusting for stroke volume**					
**Patients not censored for the start date of clinical trials and disease modifying therapy**			**Patients censored for the start date of clinical trials and disease modifying therapy**		
**Variable**	**HR (95%CI)**	**P-value**	**Variable**	**HR (95%CI)**	**P-value**
Stroke volume (ml)	0.98 (0.97-0.99)	<0.001	Stroke volume (ml)	0.98 (0.97-0.99)	<0.001
Anaemia	1.28 (1.10-1.49)	0.001	Anaemia	1.26 (1.08-1.47)	0.004
Hyponatraemia	1.68 (1.27-2.21)	<0.001	Hyponatraemia	1.63 (1.23-2.17)	0.001
Uraemia	1.43 (1.21-1.68)	<0.001	Uraemia	1.44 (1.21-1.70)	<0.001
Hyperbilirubinemia	1.22 (1.03-1.46)	0.023	Hyperbilirubinemia	1.21 (1.01-1.45)	0.041
High ALP	1.29 (1.07-1.55)	0.006	High ALP	1.25 (1.04-1.52)	0.018
High GGT	1.04 (0.86-1.25)	0.708	High GGT	1.01 (0.83-1.23)	0.908
Hypoalbuminemia	1.42 (0.85-2.34)	0.177	Hypoalbuminemia	1.47 (0.89-2.44)	0.134
High CRP	1.10 (0.94-1.28)	0.248	High CRP	1.11 (0.95-1.30)	0.200
Troponin-T >56ng/L	2.09 (1.75-2.50)	<0.001	Troponin-T >56ng/L	1.93 (1.61-2.31)	<0.001
Harrell's c	0.705 (0.686-0.724)	<0.001	Harrell's c	0.697 (0.676-0.717)	<0.001

HR = Hazard ratio; CI = Confidence interval; ALP = Alkaline phosphatase; GGT = gamma-glutamyl transferase; CRP = C-reactive protein.

Table S11. Multivariable Cox proportional hazards regression analysis of risk of death in the overall ATTR-CA population.

Multivariable Cox regression analysis for patient with a LVEF ≤40%					
Patients not censored for the start date of clinical trials and disease modifying therapy			Patients censored for the start date of clinical trials and disease modifying therapy		
**Variable**	**HR (95%CI)**	**P-value**	**Variable**	**HR (95%CI)**	**P-value**
Age (years)	1.03 (1.01-1.05)	<0.001	Age (years)	1.03 (1.01-1.05)	0.001
hATTR-CA	1.47 (1.14-1.89)	0.003	hATTR-CA	1.44 (1.10-1.87)	0.007
NAC stage 1	ref		NAC stage 1	ref	
NAC stage 2	1.46 (1.05-2.02)	0.025	NAC stage 2	1.50 (1.06-2.12)	0.022
NAC stage 3	1.73 (1.17-2.57)	0.005	NAC stage 3	1.66 (1.11-2.49)	0.015
Anaemia	1.39 (1.08-1.81)	0.012	Anaemia	1.43 (1.10-1.87)	0.008
Hyponatraemia	2.51 (1.62-3.87)	<0.001	Hyponatraemia	2.52 (1.62-3.93)	<0.001
Uraemia	0.97 (0.71-1.32)	0.851	Uraemia	0.91 (0.66-1.25)	0.562
Hyperbilirubinemia	1.18 (0.89-1.56)	0.242	Hyperbilirubinemia	1.18 (0.89-1.57)	0.260
High ALP	1.39 (1.05-1.86)	0.023	High ALP	1.34 (1.00-1.81)	0.049
High GGT	0.90 (0.79-1.32)	0.871	High GGT	0.89 (0.62-1.28)	0.546
Hypoalbuminemia	1.14 (0.49-2.64)	0.766	Hypoalbuminemia	1.24 (0.53-2.87)	0.622
High CRP	1.02 (0.79-1.32)	0.871	High CRP	1.06 (0.82-1.38)	0.638
Troponin-T>56ng/L	1.64 (1.18-2.29)	0.003	Troponin-T>56ng/L	1.72 (1.22-2.44)	0.002
Harrell's c	0.709 (0.676-0.740)	<0.001	Harrell's c	0.712 (0.79-0.745)	<0.001
**Multivariable Cox regression analysis for patient with a LVEF 40%**					
**Patients not censored for the start date of clinical trials and disease modifying therapy**			**Patients censored for the start date of clinical trials and disease modifying therapy**		
**Variable**	**HR (95%CI)**	**P-value**	**Variable**	**HR (95%CI)**	**P-value**
Age (years)	1.02 (1.01-1.04)	<0.001	Age (years)	1.02 (1.01-1.03)	0.004
hATTR-CA	1.58 (1.31-1.91)	<0.001	hATTR-CA	1.56 (1.28-1.91)	<0.001
NAC stage 1	ref		NAC stage 1	ref	
NAC stage 2	1.58 (1.30-1.93)	<0.001	NAC stage 2	1.57 (1.27-1.93)	<0.001
NAC stage 3	1.94 (1.51-2.50)	<0.001	NAC stage 3	2.08 (1.60-2.70)	<0.001
Anaemia	1.11 (0.94-1.31)	0.206	Anaemia	1.08 (0.91-1.29)	0.368
Hyponatraemia	1.43 (1.04-1.96)	0.028	Hyponatraemia	1.51 (1.08-2.10)	0.015
Uraemia	1.33 (1.09-1.61)	0.004	Uraemia	1.37 (1.12-1.68)	0.002
Hyperbilirubinemia	1.39 (1.13-1.72)	0.002	Hyperbilirubinemia	1.35 (1.08-1.69)	0.007
High ALP	1.06 (0.85-1.32)	0.604	High ALP	1.06 (0.85-1.69)	0.594
High GGT	1.15 (0.94-1.40)	0.166	High GGT	1.14 (0.92-1.40)	0.227
Hypoalbuminemia	1.95 (1.18-3.22)	0.009	Hypoalbuminemia	1.93 (1.16-3.20)	0.011
High CRP	1.19 (1.00-1.42)	0.054	High CRP	1.15 (0.96-1.38)	0.123
Troponin-T>56ng/L	1.72 (1.42-2.09)	<0.001	Troponin-T>56ng/L	1.61 (1.31-1.97)	<0.001
Harrell's c	0.719 (0.699-0.40)	<0.001	Harrell's c	0.714 (0.693-0.736)	<0.001

LVEF = Left ventricular ejection fraction; hATTR-CA = Hereditary transthyretin cardiac amyloidosis; NAC = National Amyloidosis Centre; HR = Hazard ratio; CI = Confidence interval; ALP = Alkaline phosphatase; GGT = gamma-glutamyl transferase; CRP = C-reactive protein.
